# Urea cycle fumarate limits fibrosis post-myocardial infarction by reducing fibroblast mitochondrial adenosine triphosphate production

**DOI:** 10.1093/cvr/cvag119

**Published:** 2026-05-28

**Authors:** Jing Zhao, Yating Ruan, Yongjian Chen, Qiming Chen, Tingting Hong, Linjun Wang, Yinghui Xu, Liya Hou, Fei Liao, Deling Yin, Cheng Ni

**Affiliations:** Department of Cardiology of the Second Affiliated Hospital, School of Medicine, Zhejiang University, 88# Jiefang Road, Hangzhou, Zhejiang Province 310009, China; State Key Laboratory of Transvascular Implantation Devices, 88# Jiefang Road, Hangzhou, Zhejiang Province 310009, China; Heart Regeneration and Repair Key Laboratory of Zhejiang Province, 88# Jiefang Road, Hangzhou, Zhejiang Province 310009, China; Transvascular Implantation Devices Research Institute, Binjiang Institute of Zhejiang University, Building 2, Huoju Innovation Center, No. 239 Jucai Road, Changhe Subdistrict, Binjiang District, Hangzhou, Zhejiang Province 310053, China; Department of Cardiology of the Second Affiliated Hospital, School of Medicine, Zhejiang University, 88# Jiefang Road, Hangzhou, Zhejiang Province 310009, China; State Key Laboratory of Transvascular Implantation Devices, 88# Jiefang Road, Hangzhou, Zhejiang Province 310009, China; Heart Regeneration and Repair Key Laboratory of Zhejiang Province, 88# Jiefang Road, Hangzhou, Zhejiang Province 310009, China; Transvascular Implantation Devices Research Institute, Binjiang Institute of Zhejiang University, Building 2, Huoju Innovation Center, No. 239 Jucai Road, Changhe Subdistrict, Binjiang District, Hangzhou, Zhejiang Province 310053, China; Department of Cardiology, The First Affiliated Hospital of Wenzhou Medical University, Wenzhou 325000, China; Department of Cardiology of the Second Affiliated Hospital, School of Medicine, Zhejiang University, 88# Jiefang Road, Hangzhou, Zhejiang Province 310009, China; State Key Laboratory of Transvascular Implantation Devices, 88# Jiefang Road, Hangzhou, Zhejiang Province 310009, China; Heart Regeneration and Repair Key Laboratory of Zhejiang Province, 88# Jiefang Road, Hangzhou, Zhejiang Province 310009, China; Transvascular Implantation Devices Research Institute, Binjiang Institute of Zhejiang University, Building 2, Huoju Innovation Center, No. 239 Jucai Road, Changhe Subdistrict, Binjiang District, Hangzhou, Zhejiang Province 310053, China; Department of Cardiology of the Second Affiliated Hospital, School of Medicine, Zhejiang University, 88# Jiefang Road, Hangzhou, Zhejiang Province 310009, China; State Key Laboratory of Transvascular Implantation Devices, 88# Jiefang Road, Hangzhou, Zhejiang Province 310009, China; Heart Regeneration and Repair Key Laboratory of Zhejiang Province, 88# Jiefang Road, Hangzhou, Zhejiang Province 310009, China; Transvascular Implantation Devices Research Institute, Binjiang Institute of Zhejiang University, Building 2, Huoju Innovation Center, No. 239 Jucai Road, Changhe Subdistrict, Binjiang District, Hangzhou, Zhejiang Province 310053, China; Department of Cardiology of the Second Affiliated Hospital, School of Medicine, Zhejiang University, 88# Jiefang Road, Hangzhou, Zhejiang Province 310009, China; State Key Laboratory of Transvascular Implantation Devices, 88# Jiefang Road, Hangzhou, Zhejiang Province 310009, China; Heart Regeneration and Repair Key Laboratory of Zhejiang Province, 88# Jiefang Road, Hangzhou, Zhejiang Province 310009, China; Transvascular Implantation Devices Research Institute, Binjiang Institute of Zhejiang University, Building 2, Huoju Innovation Center, No. 239 Jucai Road, Changhe Subdistrict, Binjiang District, Hangzhou, Zhejiang Province 310053, China; Department of Cardiology of the Second Affiliated Hospital, School of Medicine, Zhejiang University, 88# Jiefang Road, Hangzhou, Zhejiang Province 310009, China; State Key Laboratory of Transvascular Implantation Devices, 88# Jiefang Road, Hangzhou, Zhejiang Province 310009, China; Heart Regeneration and Repair Key Laboratory of Zhejiang Province, 88# Jiefang Road, Hangzhou, Zhejiang Province 310009, China; Transvascular Implantation Devices Research Institute, Binjiang Institute of Zhejiang University, Building 2, Huoju Innovation Center, No. 239 Jucai Road, Changhe Subdistrict, Binjiang District, Hangzhou, Zhejiang Province 310053, China; Department of Cardiology of the Second Affiliated Hospital, School of Medicine, Zhejiang University, 88# Jiefang Road, Hangzhou, Zhejiang Province 310009, China; State Key Laboratory of Transvascular Implantation Devices, 88# Jiefang Road, Hangzhou, Zhejiang Province 310009, China; Heart Regeneration and Repair Key Laboratory of Zhejiang Province, 88# Jiefang Road, Hangzhou, Zhejiang Province 310009, China; Transvascular Implantation Devices Research Institute, Binjiang Institute of Zhejiang University, Building 2, Huoju Innovation Center, No. 239 Jucai Road, Changhe Subdistrict, Binjiang District, Hangzhou, Zhejiang Province 310053, China; Department of Cardiology of the Second Affiliated Hospital, School of Medicine, Zhejiang University, 88# Jiefang Road, Hangzhou, Zhejiang Province 310009, China; State Key Laboratory of Transvascular Implantation Devices, 88# Jiefang Road, Hangzhou, Zhejiang Province 310009, China; Heart Regeneration and Repair Key Laboratory of Zhejiang Province, 88# Jiefang Road, Hangzhou, Zhejiang Province 310009, China; Transvascular Implantation Devices Research Institute, Binjiang Institute of Zhejiang University, Building 2, Huoju Innovation Center, No. 239 Jucai Road, Changhe Subdistrict, Binjiang District, Hangzhou, Zhejiang Province 310053, China; Department of Cardiology of the Second Affiliated Hospital, School of Medicine, Zhejiang University, 88# Jiefang Road, Hangzhou, Zhejiang Province 310009, China; State Key Laboratory of Transvascular Implantation Devices, 88# Jiefang Road, Hangzhou, Zhejiang Province 310009, China; Heart Regeneration and Repair Key Laboratory of Zhejiang Province, 88# Jiefang Road, Hangzhou, Zhejiang Province 310009, China; Transvascular Implantation Devices Research Institute, Binjiang Institute of Zhejiang University, Building 2, Huoju Innovation Center, No. 239 Jucai Road, Changhe Subdistrict, Binjiang District, Hangzhou, Zhejiang Province 310053, China; Department of Cardiology of the Second Affiliated Hospital, School of Medicine, Zhejiang University, 88# Jiefang Road, Hangzhou, Zhejiang Province 310009, China; State Key Laboratory of Transvascular Implantation Devices, 88# Jiefang Road, Hangzhou, Zhejiang Province 310009, China; Heart Regeneration and Repair Key Laboratory of Zhejiang Province, 88# Jiefang Road, Hangzhou, Zhejiang Province 310009, China; Transvascular Implantation Devices Research Institute, Binjiang Institute of Zhejiang University, Building 2, Huoju Innovation Center, No. 239 Jucai Road, Changhe Subdistrict, Binjiang District, Hangzhou, Zhejiang Province 310053, China

**Keywords:** Cardiac fibrosis, Myocardial infarction, Arginine biosynthesis, Urea cycle, Fumarate

## Abstract

**Aims:**

Cardiac fibrosis, a common pathological outcome of various heart diseases including myocardial infarction (MI), is primarily driven by the activation and trans-differentiation of cardiac fibroblasts that demand substantial adenosine triphosphate production (ATP) for energy. Although sodium-glucose co-transporter 2 (SGLT2) inhibitors such as dapagliflozin have been shown to improve outcomes in heart failure, their direct impact on cardiac fibrosis, particularly through the modulation of fibroblast energy metabolism remains unexplored.

**Methods and results:**

We employed an integrated strategy combining metabolomics and metabolic flux analysis to investigate metabolic reprogramming in cardiac fibroblasts under ischaemic conditions. Our findings confirmed that treatment with an SGLT2 inhibitor confers anti-fibrotic benefits post-MI. Multi-omics analysis identified a key metabolic pathway modulated in fibroblasts from SGLT2 inhibitor-treated mice under ischaemia: the conversion of *N*-acetyl-glutamate (NAcGlu) to fumarate, catalysed by argininosuccinate lyase (ASL). This pathway serves as a metabolic bridge linking the urea cycle to the tricarboxylic acid (TCA) cycle. Exogenous supplementation with either NAcGlu or fumarate significantly improved cardiac function and reduced fibrosis after MI. In contrast, targeted deletion of ASL in activated cardiac fibroblasts impaired cardiac performance, even with NAcGlu supplementation. Mechanistically, we found that fumarate accumulation under stress presses the TCA cycle in cardiac fibroblasts, resulting in reduced ATP production.

**Conclusion:**

These findings identify the NAcGlu/ASL/fumarate axis as an important regulator of fibroblast metabolism and trans-differentiation during ischaemic stress. Our data are consistent with a model in which targeting key metabolites (NAcGlu, fumarate) or enzymes (ASL) in the urea cycle pathway of cardiac fibroblasts may point to a potential therapeutic strategy to combat adverse cardiac fibrosis following MI.

## Graphical Abstract

**Graphical Abstract:**
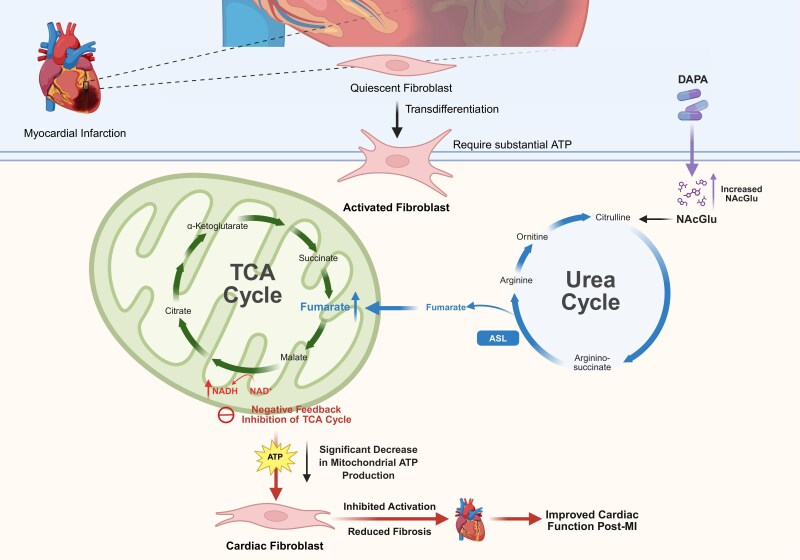

**Time of primary review: 35 days**

**See the editorial comment for this article ‘Urea-cycle control of fibroblast energetics after myocardial infarction’, by R.A. Zaki and A. El-Osta, https://doi.org/10.1093/cvr/cvag127.**

## 1. Introduction

Myocardial infarction (MI) is a refractory disease that imposes a significant economic burden on society and poses a serious threat to human health.^1^ Following MI, fibroblasts replace necrotic myocardial cells and gradually form scar tissue, a process known as cardiac fibrosis. In the early stages of MI, cardiac fibrosis plays a reparative role. However, excessive fibrosis in the later stages can lead to stiffening of the ventricular wall, progressive deterioration of cardiac function, and ultimately, heart failure.^2–5^ Substantial evidence has demonstrated that cardiac fibrosis resulting from excessive post-MI remodelling is a major contributor to impaired cardiac function and poor clinical outcomes.^6,7^ Therefore, gaining a deeper understanding of cardiac fibrosis and identifying effective interventions post-MI remain critical scientific challenges in cardiovascular research. The trans-differentiation of fibroblasts and their secretion of collagen are key processes in fibrosis, which require substantial amounts of ATP as an energy source.^8–11^ As essential stromal cells in the heart, cardiac fibroblasts serve as both initiators of cardiac fibrosis and primary producers of the extracellular matrix.^2,12,13^ Moreover, there is a strong association between fibroblast trans-differentiation and their energy metabolism, which may represent a potential therapeutic target for post-MI cardiac fibrosis.^8–11^

SGLT2 inhibitors, such as dapagliflozin (DAPA), are novel antidiabetic drugs that function by inhibiting renal glucose reabsorption.^14^ SGLT2 inhibitors have demonstrated remarkable cardiovascular benefits in large-scale clinical trials, including a significant reduction in heart failure hospitalizations in both diabetic and non-diabetic patients.^15–18^ Growing evidence suggests that the mechanisms underlying these benefits may involve direct actions on cardiac cells, including the modulation of cellular energy metabolism.^19–21^ For instance, DAPA has been shown to influence mitochondrial function and reduce oxidative stress in cardiomyocytes.^22^ Furthermore, emerging research indicates that DAPA can alter the gene expression profiles of macrophages, attenuating inflammation and fibrosis in models of chronic heart failure.^23^ Despite these advances, the specific impact of DAPA on the bioenergetics of cardiac fibroblast and the exact metabolic pathways involved remain unexplored.

Mitochondria are the primary source of ATP in cellular energy metabolism. The tricarboxylic acid (TCA) cycle within mitochondria is a central hub of energy production. Notably, the TCA cycle interfaces with other metabolic pathways, such as the urea cycle. A key metabolite at this intersection is fumarate, which can be generated from L-argininosuccinate via argininosuccinate lyase (ASL), creating a potential link between urea cycle activity and ATP production. Studies have revealed that maintaining mitochondrial homeostasis and regulating mitochondrial energy metabolism can exert protective effects by preventing excessive cardiac remodelling and heart failure.^24,25^

In this study, we unveil a novel potential metabolic pathway through which DAPA modulates fibroblast energetics to exert its anti-fibrotic effect post-MI, highlighting a potential therapeutic target for cardiac fibrosis. A limitation of this study is the lack of a direct fibroblast-specific metabolic rescue experiment, which is acknowledged as a constraint on establishing definitive causality. Future studies employing inducible genetic models or targeted metabolite delivery systems will be valuable to conclusively establish the sufficiency and necessity of this pathway.

## 2. Methods

### 2.1 Animal experiments and ethics statement

All animal studies complied with the Declaration of Helsinki and were approved by the Institutional Animal Research Committee of Zhejiang University (No. 2019-177) and were conducted in accordance with the Guide for the Care and Use of Laboratory Animals published by the U.S. National Institutes of Health.

All rats were obtained from the Zhejiang Experimental Animal Centre (Hangzhou, China). Male Sprague-Dawley rats aged 6–8 weeks were housed in a pathogen-free, temperature-controlled facility with a 12:12-h light–dark cycle. AAV9 vectors carrying the periostin promoter (*Postn*-promoter) with either NC or ASL-KD were injected into the normal LV wall under ultrasound guidance two weeks prior to MI surgery. The rats were anaesthetized with 2% isoflurane. A left parasternal incision (approximately 2 cm in length) was made along the 4th intercostal space of the left chest. Subcutaneous tissue was bluntly dissected to expose the intercostal space, and intercostal muscles were carefully transected with ophthalmic scissors to avoid injury to intercostal blood vessels and nerves. A retractor was inserted to expand the surgical field, followed by a gentle incision of the parietal pleura to access the thoracic cavity. The left anterior descending coronary artery (LAD) was identified. Using a 6–0 polypropylene suture, the LAD was ligated approximately 2–3 mm below the left atrial appendage with a slipknot. The tightness of the knot was adjusted to ensure complete arterial occlusion, which was confirmed by immediate blanching of myocardial tissue in the perfused area. For the sham-operated group, the suture was passed under the LAD without ligation. After confirming hemostasis (no active bleeding in the thoracic cavity), the lung was repositioned to ensure full expansion. Intercostal muscles and the thoracic wall were sutured layer by layer with 5–0 silk suture, while subcutaneous tissue and skin were closed with continuous sutures. During the closure, air was gently squeezed out of the thoracic cavity to prevent pneumothorax.

Experiments were performed to (i) assess the central role of the SGLT2i inhibitor DAPA (10 mg/kg) in MI and (ii) determine whether ASL knockdown inhibits the therapeutic effects of DAPA post-MI. DAPA [10 mg/kg (body weight)] or NAcGlu [5 g/kg (body weight)] was administered orally immediately by adding to the daily feed after MI induction. For the first objective, rats were randomly assigned to three groups (*n* = 15 per group): (i) Sham group, (ii) MI group, and (iii) MI with DAPA treatment (MI + SGLT2i group). For the second objective, five groups of rats (*n* = 8 per group) were studied: (i) Sham group; (ii) MI group with periostin promoter ASL-NC (MI + *Postn*-promoter ASL-NC); (iii) MI group with periostin promoter ASL-NC and DAPA treatment (MI + *Postn*-promoter ASL-NC + SGLT2i); (iv) MI group with periostin promoter ASL knockdown (MI + *Postn*-promoter ASL-KD); (v) MI group with periostin promoter ASL knockdown and DAPA treatment (MI + *Postn*-promoter ASL-KD + SGLT2i). High-frequency echocardiography was performed at baseline (day −1), after MI but before treatment (day 3) and post-treatment (day 7, 14, and 28). On day 28, the rats were euthanized and their blood and heart tissues were collected for analysis. Adult rats were euthanized using 3% isoflurane inhalation followed by cervical dislocation. The neonatal rats were euthanized using 3% isoflurane inhalation followed by decapitation.

### 2.2 Human heart tissue sample and ethics statement

Tissue samples were taken from the free wall of the left ventricle from two types of donated hearts: one type was derived from individuals without organic lesions or brain-dead patients (serving as the control group), and the other type was derived from patients who developed MI-related heart failure and subsequently underwent heart transplantation. All procedures were approved by the Ethics Review Committee of the Second Affiliated Hospital of Zhejiang University. All subjects were fully informed and signed a consent by patients or their relatives before sample collection. To isolate cardiac fibroblasts, hearts were excised and rinsed in cold Hank's balanced salt solution. The tissue was minced into 1 mm^3^ pieces and digested at 37°C for 15 min using type II collagenase (100 U/mL; Worthington, USA) and pancreatin (0.6 mg/mL; Sigma, USA). After each digestion, the supernatant was collected and resuspended in Dulbecco's modified Eagle's medium (DMEM—Gibco, Thermo Fisher Scientific, USA) supplemented with 10% foetal bovine serum (Thermo Fisher Scientific) and 1% antibiotic solution. The digestion process was repeated (typically six times) until the digestion fluid became clear. All supernatants were pooled and centrifuged at 600 *g* for 10 min. The resulting cell pellet was plated onto 100 mm culture dishes.

### 2.3 Adeno-associated virus construction and infection

Briefly, AAV9 carrying a *postn-*promoter driving the expression of shRNA targeting ASL (AAV9-*postn-*promoter-shASL) was constructed by Shanghai Genechem Co. (Shanghai, China). The shRNA sequence for ASL was CCAAGGAAUUCAACUUUGUTT. Either AAV9-*postn-*promoter-shASL or AAV9-*postn-*promoter-shScramble (1E + 12 v.g./mL) was injected into the normal LV wall 2 weeks prior to MI surgery, under ultrasound guidance (see Supplementary material online, Figure  *S9A*).

### 2.4 2D Echocardiography on rats

Transthoracic two-dimensional echocardiography was performed using a 13–24 MHz transducer (Vevo 3100 Imaging System, VisualSonics, FUJIFILM, Canada) on rats anaesthetized via inhalation of isoflurane (1.5–2%) (RWD, China). B-mode and M-mode imaging were performed in the parasternal long-axis view at the level of the greatest LV end-diastolic dimension. Left ventricular dimensions, including diastolic (LVPW;d) and systolic (LVPW;s) posterior wall thickness and internal diameters at end-diastole (LVID;d) and end-systole (LVID;s), were measured. Left ventricular ejection fraction (EF) and fractional shortening (FS) were automatically calculated using the integrated echocardiographic software (Vevo LAB 5.8.1).

### 2.5 Quasi-targeted metabolomics

Neonatal rat cardiac fibroblasts (NRCFs) were seeded in 6 cm dishes at a density of 2 × 10^6^ cells and cultured to 80% confluence. Cells were then treated with transforming growth factor beta (TGF-β, 10 ng/mL) and/or DAPA (10 μM) for 24 h. After treatment, cells were freeze-dried and resuspended in pre-chilled 80% methanol, followed by thorough vortex. Samples were incubated on ice for 5 min and subjected to three cycles of liquid nitrogen lysis. Lysates were centrifuged at 15 000 *g*, 4°C for 15 min. A portion of the supernatant was diluted with liquid chromatography–tandem mass spectrometry (LC–MS) grade water to a final concentration of 80% methanol. Samples were transferred to fresh Eppendorf tubes and centrifuged again at 15 000 *g*, 4°C for 15 min. Finally, the supernatant was used for LC–MS/MS system analysis.

### 2.6 ^13^C-Labelled targeted metabolic flux analysis

For ^13^C-carbon incorporation from [U-^13^C] glucose in metabolic flux analysis, NRCFs were seeded in 6 cm dishes at a density of 5 × 10^6^ cells and cultured to 80% confluence. Cells were then exposed to TGF-β (10 ng/mL) and/or DAPA (10 μM) for 24 h. After treatment, cells were washed with phosphate buffered saline (PBS) and the labelled substrate, [U-^13^C_6_] glucose (CLM-1396-1, Cambridge Isotope Laboratories, USA), was added for 4 h to confirm steady-state labelling. Following incubation, cells were washed twice with cold 0.9% saline, and 500 μL of cold extraction buffer (methanol:acetonitrile:water = 2:2:1, v/v/v) was added to each sample. Samples were subjected to three cycles of liquid nitrogen lysis and then centrifuged at 14 000 *g* for 5 min at 4°C.

### 2.7 Seahorse assay

The XF96 extracellular flux analyser (Seahorse Bioscience) was used to measure the extracellular acidification rate (ECAR), oxygen consumption rate (OCR), and ATP real-time rate. Cells were seeded in Seahorse 96-well assay plates at a density of 2 × 10^4^ cells/well for NRCFs, 1 × 10^4^ cells/well for ARCFs (adult rat cardiac fibroblast) and incubated overnight, respectively. The sensor cartridge was hydrated at 37°C in a CO_2_-free incubator overnight. ECAR was assessed using the ECAR determination kit (103020-100, Agilent Technologies, USA), while OCR was measured using the OCR determination kit (103015-100, Agilent Technologies, USA). The ATP real-time rate was determined using the Seahorse XF96 real-time ATP rate assay kit (103592-100, Agilent Technologies, USA). All measurements were normalized to the total protein concentration in each well. Each assay was performed in triplicate for each condition.

### 2.8 Statistical analysis

Quantitative data are presented as mean ± S.E.M., based on results from at least three independent experimental replicates. The normality of data distribution was assessed using the Shapiro–Wilk test or Kolmogorov–Smirnov test. Statistical differences between the two groups were evaluated using a two-tailed unpaired Student's *t*-test. For comparisons involving three or more groups, one-way analysis of variance (ANOVA) was performed, followed by Tukey's *post hoc* test for multiple pairwise comparisons. For non-normally distributed data, the Kruskal–Wallis test was used for multiple group comparisons. Categorical variables were expressed as counts and percentages and compared using the chi-square test. All statistical analyses were conducted using GraphPad version 10.0. A *P-*value <0.05 was considered statistically significant.

Detailed methods are available in Supplementary material online.

## 3. Results

### 3.1 DAPA alleviates cardiac fibrosis by inhibiting fibroblast mitochondrial ATP production post-MI

**Figure 1:**
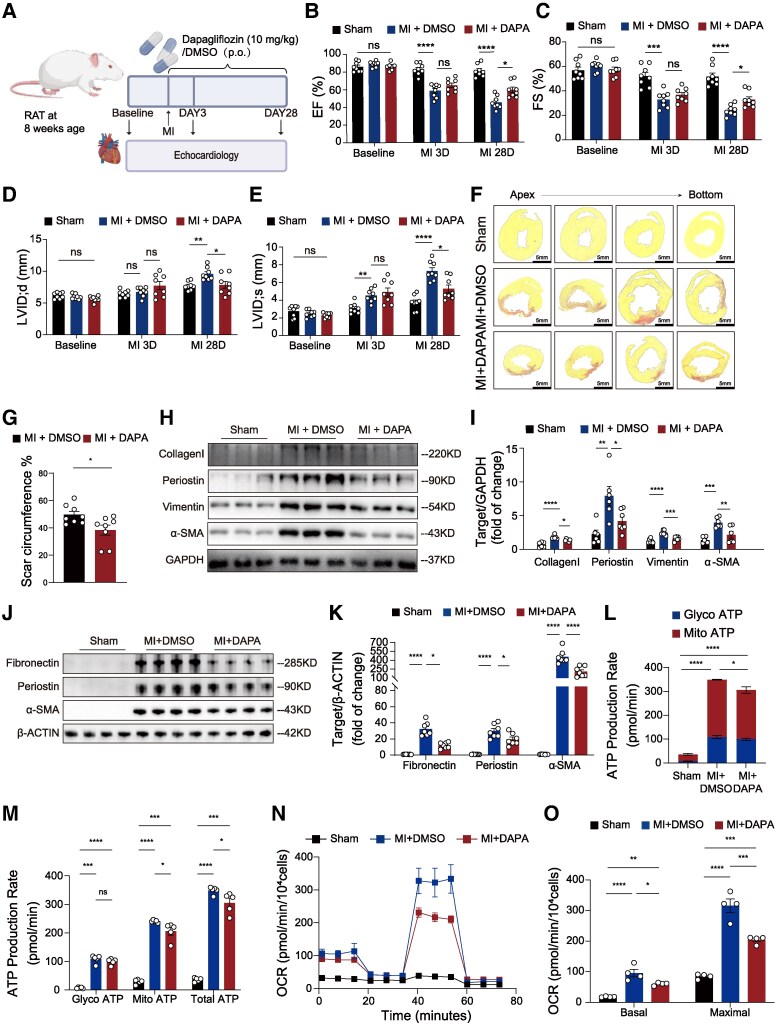
DAPA attenuates cardiac fibrosis post-MI and reduces fibroblast mitochondrial ATP production. (*A*) Schematic representation of the experimental design in rats with acute myocardial infarction (AMI) treated with or without DAPA. (*B–E*) Quantitative echocardiographic analysis; *n* = 8 per group. *ns*, not significant; *P < 0.05, **P < 0.01, ***P < 0.001， ****P < 0.0001. (*F*) Representative images of Picro-Sirius red-stained heart sections from each experimental group. Scale bar = 5 mm. (*G*) Quantification of scar circumference ratio based on Picro-Sirius red staining; *n* = 8 per group. *P < 0.05. (*H*) Expression of fibrotic markers—collagen I, periostin, vimentin, and α-SMA, evaluated in sham-operated and AMI rats treated with dimethyl sulfoxide (DMSO) or DAPA 28 days post-MI. (*I*) Quantitative analysis of fibrotic protein levels; *n* = 6 per group. *P < 0.05, **P < 0.01, ***P < 0.001， ****P < 0.0001. (*J*) Detection of fibrotic proteins in ARCFs isolated from Sham, MI + DMSO and MI + DAPA groups. (*K*) Quantitative analysis of fibrotic protein expression; *n* = 7 per group. (*L*) ATP production rate measured using a Seahorse XFe extracellular flux analyser. The statistical results were derived from the mito ATP in each group, *P < 0.05， ****P < 0.0001. (*M*) Quantification of ATP production derived from glycolysis (glyco ATP) and mitochondrial oxidative phosphorylation (mito ATP); *n* = 5 per group. *P < 0.05, ***P < 0.001, ****P < 0.0001. (*N*) The OCR of live fibroblasts was measured by a Seahorse XFe extracellular flux analyser. (*O*) Quantitative analysis of basal and maximal respiration: *n* = 4 per group. *P < 0.05, **P < 0.01, ***P < 0.001， ****P < 0.0001. All statistical data are presented as mean ± standard error of the mean (S.E.M.). Two-way ANOVA followed by Tukey's *post hoc* test was used for statistical analysis in (*B–E*). One-way ANOVA followed by Tukey's *post hoc* test was used in (*G*), (*I*), (*K*), (*M*), and (*O*). *ns*, not significant; *P < 0.05, **P < 0.01, ***P < 0.001， ****P < 0.0001.

To investigate the therapeutic potential of DAPA after MI, we subjected rats to MI surgery followed immediately by daily oral administration of DAPA (10 mg/kg) or vehicle control (*Figure 1A*). Echocardiographic assessment at days 3 and 28 post-MI revealed that DAPA treatment significantly improved cardiac function, evidenced by increased EF and FS, alongside reduced left ventricular internal dimensions during both systole and diastole (LVID;s, LVID;d) (*Figure 1B–E*, Supplementary material online, Figure  *S1A*). Haemodynamic studies corroborated these cardio-protective effects of DAPA (see Supplementary material online, Figure  *S1B*). Consistent with the functional improvement, DAPA treatment markedly attenuated cardiac fibrosis. Histological analyses using Sirius red and wheat germ agglutinin (WGA) staining showed a substantial reduction in scar size and fibrotic area in both the infarct and border zones of DAPA-treated hearts (*Figure 1F* and *G*, Supplementary material online, Figure  *S1C*–*H*, *I*, and *J*). At the molecular level, western blotting confirmed a decrease in collagen deposition and the expression of key pro-fibrotic proteins, including collagen I, periostin, and vimentin (*Figure 1H* and *I*). However, DAPA treatment given to rats began from 14 and 28 days after MI and showed moderate improvement in cardiac function and fibrosis, but did not achieve statistical significance according to the above mentioned therapeutic effects (see Supplementary material online, Figure  *S2A*–*J*). Taken together, these data establish that DAPA ameliorates cardiac remodelling and dysfunction post-MI by suppressing fibrosis.

In addition to its role in alleviating cardiac fibrosis, we next investigated whether the benefits of DAPA extended to other cardiac cell types. However, assessment of cardiomyocyte apoptosis, immune cell infiltration, and endothelial cell function revealed no significant effects under DAPA treatment (see Supplementary material online, Figure  *S3A*–*S*). These results indicate that the cardio-protective effect of DAPA is predominantly mediated through a direct action on cardiac fibroblasts.

Based on the above *in vivo* findings, we hypothesized that DAPA directly inhibits cardiac fibroblast activation. To confirm, we collected rat cardiac fibroblasts via flow sorting in rats suffering from MI injury to induce activation, in the presence or absence of DAPA. As illustrated in *Figure 1J* and *K*, DAPA also significantly suppressed the expression of pro-fibrotic proteins induced by MI injury. Given the intimate links between cellular metabolism and fibrotic responses, we profiled the bioenergetic status of fibroblasts. Seahorse analysis revealed that MI injury significantly boosted mitochondrial ATP production and OCR, both of which were effectively normalized by DAPA treatment (*Figure 1L–O*). In contrast, ECAR, an indicator of glycolysis, was unaffected (see Supplementary material online, Figure  *S4A* and *B*). Similar results were shown on NRCFs with TGF-β stimulation to simulate the *in vivo* environment of MI (see Supplementary material online, Figure  *S4C*–*J*). Collectively, these findings demonstrate that DAPA inhibits cardiac fibroblast activation by modulating mitochondrial ATP production without altering glycolysis in the context of MI.

Since fibroblast proliferation is also related to fibrosis procedure and requires ATP, we next tested proliferative related signalling like p-P38 and p-ERK and observed a reduction of their phosphorylation forms after DAPA treatment at tissue level 7 days after MI (see Supplementary material online, Figure  *S5A* and *B*). In addition, we observed through Ki67 staining at 3 and 7 days after MI that DAPA treatment suppressed fibroblast proliferation (see Supplementary material online, Figure  *S5C*–*H*). Besides, there was no evidence indicated that DAPA treatment would influence fibroblasts migration (see Supplementary material online, Figure  *S5I* and *J*). In summary, we discovered that DAPA treatment alleviates cardiac fibrosis through reducing ATP production in cardiac fibroblasts under ischaemic condition.

### 3.2 *N*-acetyl-glutamate modulates cardiac fibrosis *via* regulating fibroblast energy metabolism

**Figure 2:**
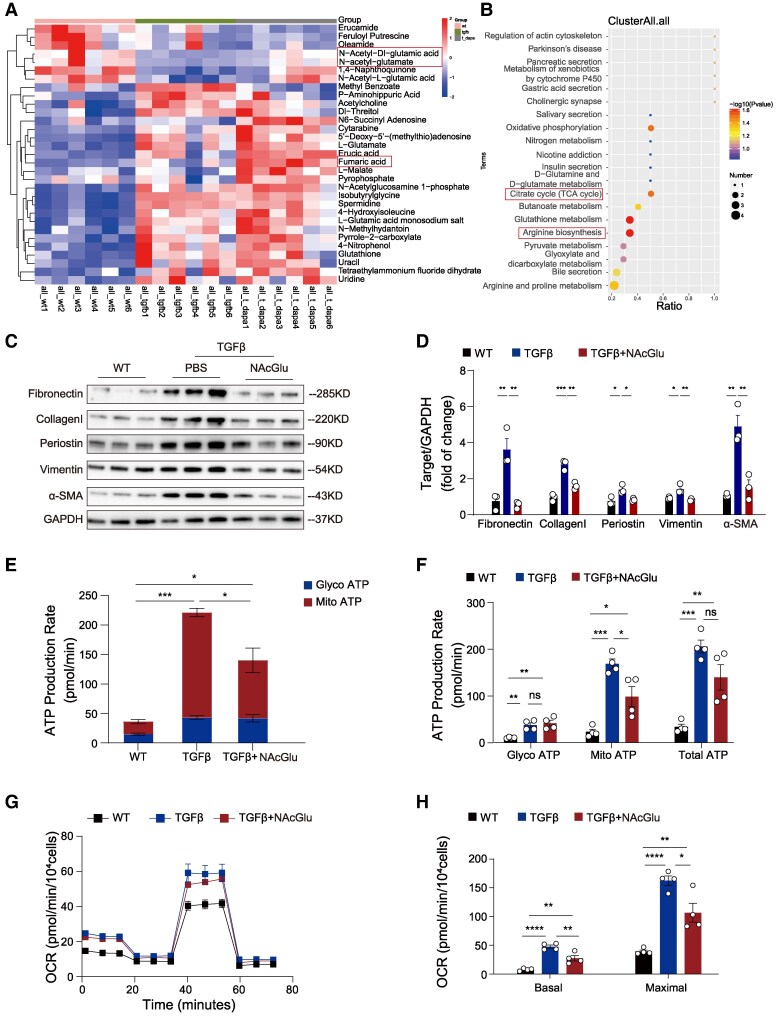
*NAcGlu* regulates fibroblast trans-differentiation and energy metabolism. (*A*) Heatmap showing metabolic profiles of NRCFs under wild-type conditions or stimulated with TGF-β, with or without DAPA treatment for 24 h. (*B*) Metabolomic pathway analysis integrated with KEGG analysis identifying metabolic pathways associated with DAPA treatment. (*C*) Immunoblots of fibrotic markers in NRCFs treated with DMEM or stimulated with TGF-β, with or without NAcGlu treatment. (*D*) Quantitative analysis of fibrotic protein levels; *n* = 3 per group. (*E*) ATP production rate measured using the Seahorse XFe extracellular flux analyser. Blue indicates ECAR; red indicates OCR. The statistical results were derived from the mito ATP in each group, *P < 0.05, ***P < 0.001. (*F*) Quantitative analysis of glyco ATP and mito ATP; *n* = 4 per group. (*G*) OCR in live fibroblasts was recorded using the Seahorse XFe extracellular flux analyser. (*H*) Quantification of basal and maximal respiration; *n* = 4 in the DMEM group, *n* = 4 in the TGF-β group and *n* = 4 in the TGF-β + NAcGlu group. All statistical data are presented as mean ± S.E.M. One-way ANOVA followed by Tukey's *post hoc* test was used in (*D*), (*F*), and (*H*). ns, not significant; *P < 0.05, **P < 0.01, ***P < 0.001.

To identify the metabolic mediators underlying DAPA's effects, we performed non-targeted metabolomics on TGF-β-stimulated NRCFs with or without DAPA treatment (see Supplementary material online, Figure  *S6A*–*C*). This analysis revealed that *N*-acetyl-glutamate (NAcGlu) occupied the top rank among metabolites that was significantly suppressed by TGF-β but restored by DAPA (*Figure 2A*, Supplementary material online, Figure  *S6D*–*F*). Kyoto encyclopedia of genes and genomes (KEGG) pathway analysis further indicated that differentially expressed metabolites were enriched in arginine biosynthesis, where NAcGlu serves as an important intermediate (*Figure 2B*). This suggested that NAcGlu might serve as a key metabolite in restricting cardiac fibroblast activation.

We, therefore, evaluated whether NAcGlu exerts inhibitory effect on fibroblast activation. Indeed, exogenous NAcGlu treatment recapitulated the anti-fibrotic effects of DAPA, significantly suppressing the expression of pro-fibrotic proteins (*Figure 2C* and *D*). More importantly, NAcGlu mirrored DAPA's metabolic impact by specifically reducing mitochondrial ATP production and OCR without affecting glycolysis (*Figure 2E–H*). The anti-fibrotic and metabolic-suppressive effects were further confirmed using a structural analogue of NAcGlu, carglumic acid (CarGlu) (see Supplementary material online, Figure  *S6G*–*J*). Collectively, these data identify NAcGlu, an essential intermediate in the urea cycle, as a key metabolite whose restoration contributes to DAPA-induced inhibition of fibroblast activation by limiting mitochondrial energy production.

### 3.3 *N*-acetyl-glutamate synthase in the urea cycle catalyses the conversion of glutamate to NAcGlu to attenuate cardiac fibrosis

Having established NAcGlu as a functional metabolite, we next investigated the role of its synthesizing enzyme, *N*-acetyl-glutamate synthase (NAGS). It is well established that NAGS promotes the conversion of glutamate to NAcGlu, thereby initiating the urea cycle and maintaining essential metabolic balance in mammals. However, the role of urea cycle regulation in organ fibrosis has not been reported. To evaluate whether NAGS expression contributes to the inhibition of cardiac fibrosis, we first examined changes in NAGS expression under TGF-β stimulation and DAPA treatment. The results showed that DAPA treatment did not significantly alter NAGS expression (see Supplementary material online, Figure  *S7A* and *B*). In addition, knockdown of NAGS had no effect on cardiac fibrosis as well as ATP production (see Supplementary material online, Figure  *S7C*–*H*). Therefore, we concluded that NAGS expression does not play a central role in regulating fibroblast activation or energy metabolism.

**Figure 3:**
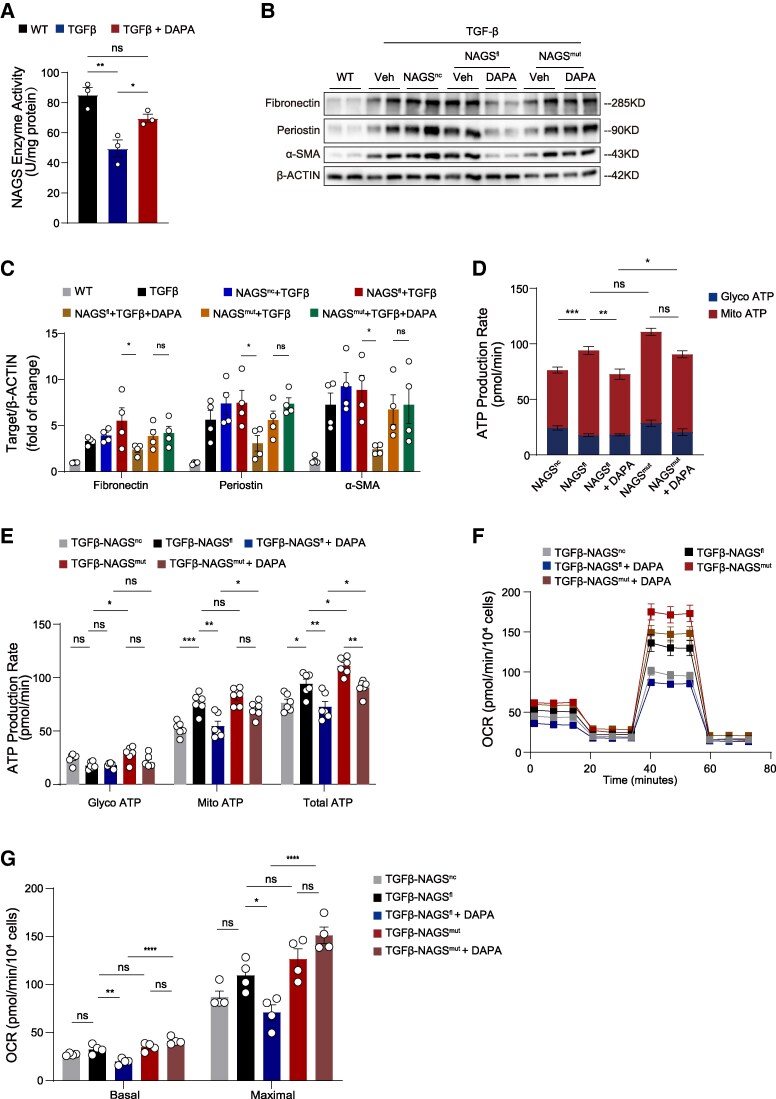
Mutation in the NAGS enzyme activity site suppresses the anti-fibrotic effect of DAPA. (*A*). NAGS enzyme activity was assessed in DMEM-treated NRCFs and TGF-β-stimulated NRCFs treated with either DMSO or DAPA using an ELISA kit; *n* = 3 per group. (*B*) Immunoblotting for fibrotic proteins was performed in NRCFs and NRCFs transfected with NAGSnc lentivirus, TGF-β-stimulated NRCFs transfected with full-length NAGS (NAGS^fl^) and/or treated with DAPA and TGF-β-stimulated NRCFs transfected with the enzyme-inactive mutant (NAGS^mut^) and/or treated with DAPA. (*C*) Quantitative bar graphs of fibrotic protein expression; *n* = 4 per group. (*D*) ATP production rate was measured using the Seahorse XFe extracellular flux analyser. Blue indicates ECAR and red indicates OCR. The statistical results were derived from the mitoATP in each group, *P < 0.05, **P < 0.01, ***P < 0.001. (*E*) Quantitative analysis of glyco ATP and mito ATP is shown. *n* = 6 for each group: TGF-β + NAGS^nc^, TGF-β + NAGS^fl^, TGF-β + NAGS^fl^ + DAPA, TGF-β + NAGS^mut^, and TGF-β + NAGS^mut^ + DAPA. (*F*) OCR of live fibroblasts was recorded and analysed using the Seahorse XFe extracellular flux analyser. (*G*) Quantitative analysis of basal and maximal respiration was plotted. *n* = 4 per group: TGF-β + NAGS^nc^, TGF-β + NAGS^fl^, TGF-β + NAGS^fl^ + DAPA, TGF-β + NAGS^mut^, and TGF-β + NAGS^mut^ + DAPA. Data are presented as mean ± S.E.M. in all statistical plots. One-way ANOVA followed by Tukey's *post hoc* multiple comparisons test was performed in panels (*A*), (*C*), (*E*), and (*G*). ns, not significant; *P < 0.05, **P < 0.01, ***P < 0.001.

Next, to determine whether the enzymatic activity of NAGS influences cardiac fibroblast activation under TGF-β stimulation, we generated lentiviral constructs expressing either full-length NAGS (NAGS^fl^) or a mutant form lacking the enzymatic activity domain (NAGS^mut^); the empty vector served as the control (NAGS^nc^). Enzymatic activity was confirmed in both NAGS^fl^ and NAGS^mut^ constructs (see Supplementary material online, Figure  *S7I*). *In vitro* studies revealed that DAPA treatment reversed the TGF-β-induced suppression of NAGS enzymatic activity, suggesting that NAGS activity may contribute to the regulation of fibroblast activation (*Figure 3A*). Western blotting further showed that NAGS^mut^ abolished the anti-fibrotic effect of DAPA, identifying the important role of NAGS enzymatic activity in modulating cardiac fibroblast behaviour (*Figure 3B* and *C*). Given its involvement in fibroblast energy metabolism, we observed that NAGS^mut^ reversed the DAPA-mediated inhibition of mitochondrial ATP production (*Figure 3D* and *E*). Similarly, mitochondrial oxygen consumption was consistent with the ATP production findings, as NAGS^mut^ led to elevated oxygen consumption despite DAPA treatment. These results strongly support the important role of NAGS enzymatic activity and its catalysed metabolite, NAcGlu, in regulating cardiac fibroblast activation under TGF-β stimulation.

### 3.4 Urea cycle-derived fumarate plays a pivotal role in regulating the TCA cycle in fibroblast under TGF-β stimulation in cardiac fibroblast

**Figure 4:**
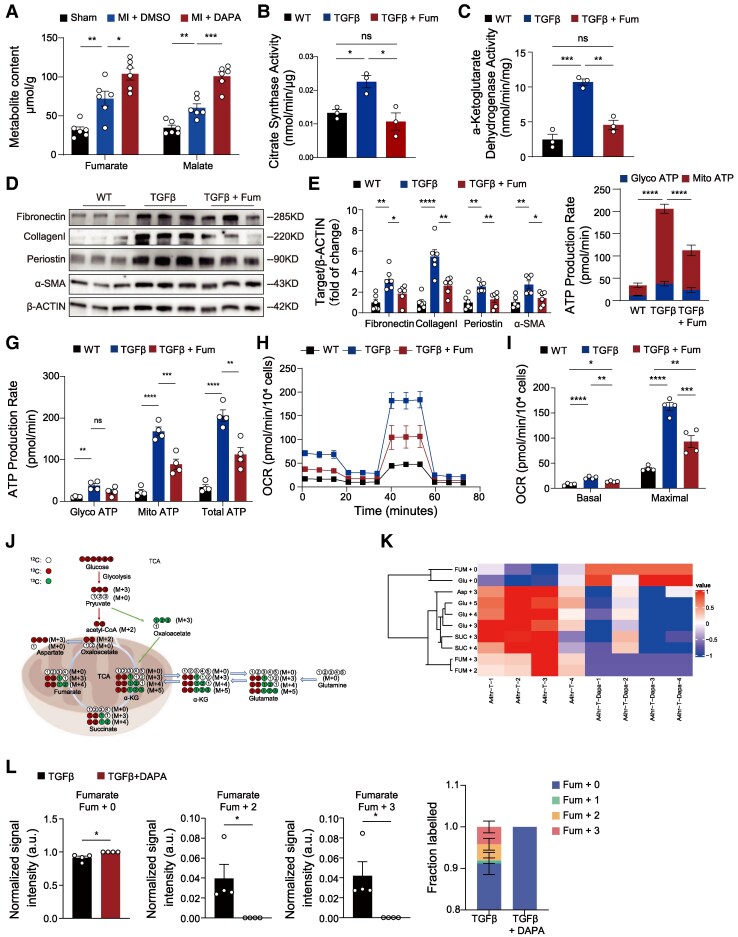
Fumarate activated by DAPA modulates fibroblasts and is regulated from urea cycle. (*A*) Fumarate and Malate content in ARCFs in Sham, MI + DMSO and MI + DAPA groups, *n* = 6 in each group. *P < 0.05, **P < 0.01, ***P < 0.001. (*B* and *C*) Activities of key enzyme CS (*B*) and α-KGDH (*C*) were measured in DMEM-treated and TGF-β-stimulated NRCFs treated with either DMSO or fumarate using an ELISA kit. *n* = 3 per group. (*D*) Expression of fibrotic proteins was detected in DMEM-treated NRCFs and TGF-β-stimulated NRCFs treated with either PBS or fumarate. (*E*) Quantitative analysis of fibrotic protein levels; *n* = 6 per group. (*F*) ATP production rate was measured using the Seahorse XFe extracellular flux analyser. The statistical results were derived from the mito ATP in each group, *P < 0.05, **P < 0.01, ***P < 0.001. (*G*) Quantitative analysis of glyco ATP and mito ATP is shown. *n* = 4 in the DMEM, TGF-β, and TGF-β + Fum groups. (*H*) OCR in live fibroblasts was recorded and analysed using the Seahorse XFe extracellular flux analyser. (*I*) Quantitative analysis of basal and maximal respiration; *n* = 4 in each of the DMEM, TGF-β, and TGF-β + Fum groups. (*J*) Schematic of the metabolism of [U-^13^C_6_] fumarate. Labelled ^13^C atoms are shown as red or green circles; unlabelled ^12^C atoms are shown as white circles. (*K*) Heatmap showing the intensity of metabolites in NRCFs after 4 h treatment with or without DAPA. (*L*) Levels of fumarate were quantified using targeted liquid chromatography–tandem mass spectrometry (LC–MS), *n* = 4 in TGF-β and TGF-β + Fum groups. *P < 0.05. Data are presented as mean ± S.E.M. in all statistical plots. One-way ANOVA followed by Tukey's *post hoc* multiple comparisons test was performed in (*A*), (*B*), (*C*), (*E*), (*G*), (*I*), and (*M*). *ns*, not significant, *P < 0.05, **P < 0.01, ***P < 0.001.

Our metabolomics analysis indicated that DAPA treatment led to the accumulation of fumarate (*Figure 2A*, Supplementary material online, Figure  *S8A*), a metabolite that bridges the urea and TCA cycles. We first asked whether compensation of fumarate could regulate TCA cycle efficiency. Thus, we collected heart tissue from rats to detect the content of metabolic products. Consistent with metabolomics analysis, we observed an increase in fumarate and downstream malate (*Figure 4A*). An increase in malic acid will cause activation in downstream reactions, leading to the accumulation of the intermediate metabolite nicotinamide adenine dinucleotide (NADH). The accumulation of NADH can inhibit the key enzymes in the TCA cycle and in turn hamper its efficiency. To verify, we assessed the activity of key TCA cycle enzymes. Fumarate treatment specifically blunted the TGF-β-induced increase in citrate synthase (CS) and α-ketoglutarate dehydrogenase (α-KGDH) activities, isocitrate dehydrogenase activity was also mildly affected but did not reach statistical significance (*Figure 4B* and *C,*  Supplementary material online, Figure  *S8B*), identifying a precise mechanism for TCA cycle inhibition. Then, we evaluated the function of fumarate on fibroblast activation. Indeed, exogenous fumarate supplementation attenuated the TGF-β-induced upregulation of fibrotic markers and, more importantly, recapitulated the suppression of mitochondrial ATP production and OCR (*Figure 4D–I*).

To definitively trace the metabolic origin of fumarate, we employed ^13^C_6_-glucose flux analysis (*Figure 4J*). As glucose is fully labelled with ^13^C isotopes, it is converted into pyruvate via glycolysis and subsequently enters the TCA cycle as acetyl-CoA. Upon entry of ^13^C-glucose into the TCA cycle through acetyl-CoA, we observed that DAPA treatment significantly increased Fum+0 and Glu+0 levels (*Figure 4K*). While DAPA treatment altered the incorporation of ^13^C into various TCA cycle intermediates (*Figure 4K*, Supplementary material online, Figure  *S8C*–*F*), the most striking finding was that nearly all cellular fumarate was not derived from the canonical TCA cycle (*Figure 4L*).

Collectively, these findings suggest that the upregulation of fumarate induced by DAPA treatment is primarily not mediated through the TCA cycle. This urea cycle-derived fumarate then acts as a break on the TCA cycle by reducing key enzyme activity, ultimately limiting the energy supply for fibroblast activation.

### 3.5 Argininosuccinate lyase plays an important role in NAcGlu-mediated fibrosis suppression by increasing fumarate content

**Figure 5:**
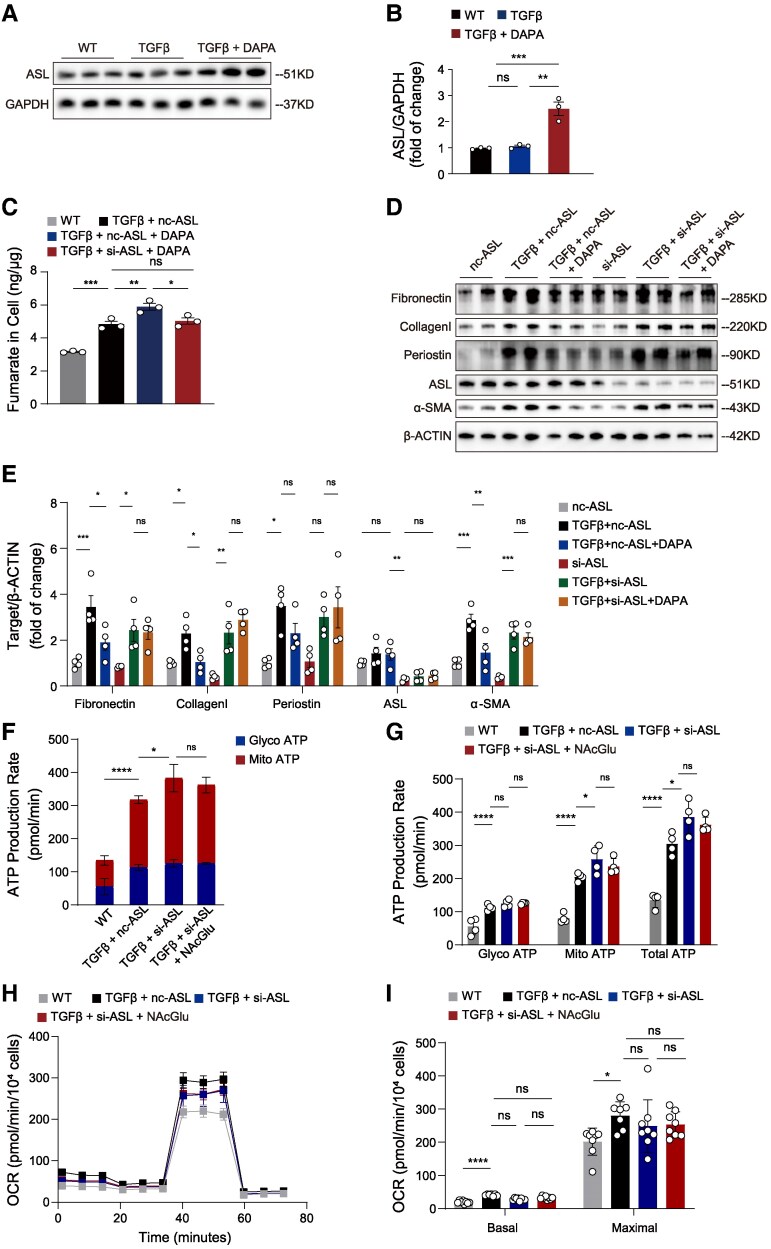
Suppression of ASL neutralizes the anti-fibrotic by DAPA. (*A*) Western-blot analysis of ASL expression in NRCFs from the DMEM, TGF-β, and TGF-β + DAPA groups. (*B*) Quantitative analysis of ASL levels shown in (*A*); *n* = 3 per group. *ns*, not significant; **P < 0.01, ***P < 0.001. (*C*) Fumarate content was measured in DMEM-treated and TGF-β-stimulated NRCFs infected with either negative control (nc-ASL) or siRNA against ASL (si-ASL) and treated with either DMSO or DAPA, using an ELISA kit. *n* = 3 per group. (*D*) Western-blot analysis of fibrotic protein and ASL expression in NRCFs from the nc-ASL, TGF-β + nc-ASL, TGF-β + nc-ASL + NAcGlu, si-ASL, TGF-β + si-ASL, and TGF-β + si-ASL + NAcGlu groups. (*E*) Quantitative analysis of related protein levels shown in (*D*); *n* = 4 per group. (*F*) ATP production rate was measured using the Seahorse XFe extracellular flux analyser. Blue represents ECAR; red represents OCR. The statistical results were derived from the mito ATP in each group, *P < 0.05, ****P < 0.0001. (*G*) Quantitative analysis of glyco ATP and mito ATP; *n* = 4 in each group, *P < 0.05, ****P < 0.0001. (*H*) OCR in live fibroblasts was recorded and analysed using the Seahorse XFe extracellular flux analyser. (*I*) Quantitative analysis of basal and maximal respiration; *n* = 8 in the DMEM group, *n* = 7 in TGF-β + nc-ASL group, *n* = 8 in TGF-β + si-ASL group, and *n* = 8 in TGF-β + si-ASL + NAcGlu group. Data are presented as mean ± S.E.M. A one-way ANOVA followed by Tukey's *post hoc* multiple comparisons test was conducted in (*B*), (*C*), (*E*), (*G*), and (*I*). *ns*, not significant, *P < 0.05, **P < 0.01, ***P < 0.001, ****P < 0.0001.

As previously mentioned, we have indicated that ASL expression is associated with fumarate levels in cardiac fibroblasts. Given the central role of fumarate accumulation in regulating the TCA cycle, we speculated that the ASL-catalysed step may link the urea cycle to the TCA cycle, ultimately associated with limited ATP production in cardiac fibroblasts. Initially, we observed that DAPA increased ASL expression at the protein level (*Figure 5A* and *B*), indicating that NAcGlu induction parallels ASL expression. As fumarate can be generated from argininosuccinate via ASL within the urea cycle, we discovered that knocking down ASL significantly reduced intracellular fumarate levels, even upon DAPA treatment (*Figure 5C*), establishing ASL as the crucial enzyme for fumarate production in this context. To further examine whether ASL activity is crucial during NAcGlu treatment in cardiac fibroblasts, we used si-ASL to suppress ASL expression (*Figure 5D* and *E*), thereby disrupting the connection between argininosuccinate and fumarate. As a result, we found that inhibiting ASL negated the anti-fibrotic effects of NAcGlu at the level of pro-fibrotic protein expression (*Figure 5F* and *G*). Moreover, NAcGlu treatment failed to reduce the elevated mitochondrial ATP production rate (*Figure 5H* and *I*) or the increased OCR (*Figure 5J* and *K*) when ASL was knocked down, compared to the control. These findings suggest that ASL contributes to the anti-fibrotic action of NAcGlu, primarily by modulating fumarate conversion.

### 3.6 Targeted intervention of ASL in cardiac fibroblasts counteracts the anti-fibrotic effects of NAcGlu post-MI

**Figure 6:**
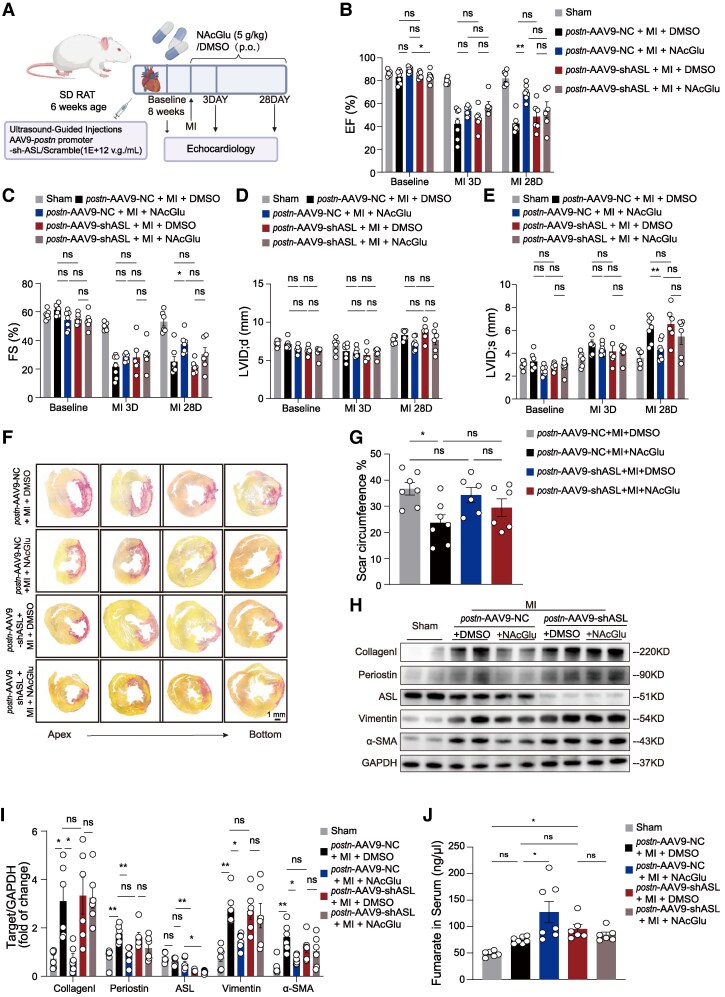
Fibroblast-specific knockdown of ASL reverses cardio-protective effect by NAcGlu under MI condition. (*A*) Schematic representation of the experimental design for NAcGlu treatment in rats receiving either *postn*-AAV9-NC-ASL or *postn*-AAV9-KD-ASL in an AMI model. (*B–E*) Quantitative analysis of echocardiographic parameters; *n* = 6–7 in each group. (*F*) Representative images of Picro-Sirius red-stained heart sections from each experimental group; scale bar = 1 mm. (*G*) Quantification of scar circumference ratio from Picro-Sirius red staining; *n* = 6–7 in each group. (*H*) Expression of fibrotic proteins, including collagen I, periostin, ASL, vimentin, and α-SMA, was assessed in sham-operated rats and AMI rats injected with *postn*-AAV9-NC-ASL or *postn*-AAV9-KD-ASL before MI, followed by PBS or NAcGlu treatment 28 days post-MI. (*I*) Quantitative analysis of fibrotic protein expression: *n* = 6–7 in each group. (*J*) Fumarate levels were measured in serum samples using an ELISA kit; *n* = 6 in the Sham group, *n* = 7 in the *postn*-AAV9-NC-ASL + MI and *postn*-AAV9-NC-ASL + MI + NAcGlu groups and *n* = 6 in the *postn*-AAV9-KD-ASL + MI and *postn*-AAV9-KD-ASL + MI + NAcGlu groups. Data are presented as mean ± S.E.M. Two-way ANOVA followed by Tukey's *post hoc* multiple comparisons test was used for (*B*), (*C*), (*D*), and (*E*). One-way ANOVA followed by Tukey's *post hoc* test was used for (*G*), (*I*), and (*J*). *ns*, not significant; *P < 0.05, **P < 0.01, ***P < 0.001.

After validating ASL as the link between the urea cycle and the TCA cycle, we next investigated whether ASL expression in cardiac fibroblasts also influences the healing process following MI. To specifically knock down ASL in cardiac fibroblasts post-MI, we generated an adeno-associated virus carrying KD-ASL driven by the *postn-*promoter to target activated fibroblasts (*postn*-AAV9-shASL); a control virus was constructed simultaneously (*postn*-AAV9-NC). Using echocardiography-guided intra-myocardial injection, we established a rat MI model with administration of either *postn*-AAV9-shASL or *postn*-AAV9-NC. In both cases, NAcGlu was also administered to evaluate its cardio-protective effects (*Figure 6A*, Supplementary material online, Figure  *S9A*). Echocardiography was performed on MI rats before MI and at day 3 and 28 post-MI. As expected, NAcGlu treatment in the *postn*-AAV9-NC group significantly attenuated MI-induced injury, as reflected by improved EF and FS compared to the *postn*-AAV9-NC control (*Figure 6B* and *C,*  Supplementary material online, Figure  *S9B*). However, the cardio-protective effect of NAcGlu was abolished in rats receiving *post*-AAV9-shASL, indicating that NAcGlu's beneficial effects depend on ASL expression post-MI. Additionally, ASL knockdown worsened NAcGlu's anti-remodelling effects, as shown by increased LVID;d and LVID;s (*Figure 6D* and *E*). To assess cardiac fibrosis, Sirius red staining of heart sections revealed that NAcGlu effectively reduced fibrotic area post-MI; however, specific ASL knockdown in cardiac fibroblasts negated these anti-fibrotic effects (*Figure 6F* and *G*). Similar findings were observed using WGA and troponin immunofluorescent staining (see Supplementary material online, Figure  *S9C* and *D*). Finally, western-blot analysis confirmed that NAcGlu's anti-fibrotic activity was inhibited by *postn*-AAV9-shASL (*Figure 6H* and *I*).

As mentioned above, we identified that NAcGlu could elevate fumarate levels to inhibit the TCA cycle, thereby restricting cardiac fibrosis. To test this, we measured serum fumarate content in rats from each group and found that NAcGlu treatment significantly increased fumarate levels, whereas ASL knockdown in cardiac fibroblasts markedly reduced this effect (*Figure 6J*). Based on these findings, we concluded that ASL mediates the conversion of NAcGlu to fumarate. The subsequent accumulation of fumarate is associated with restricted TCA cycle metabolic flux, inhibited mitochondrial ATP production and ultimately reduced cardiac fibrosis along with improved cardiac function following MI.

### 3.7 NAcGlu/ASL/fumarate metabolism functions in restricting human cardiac fibroblasts activation

**Figure 7:**
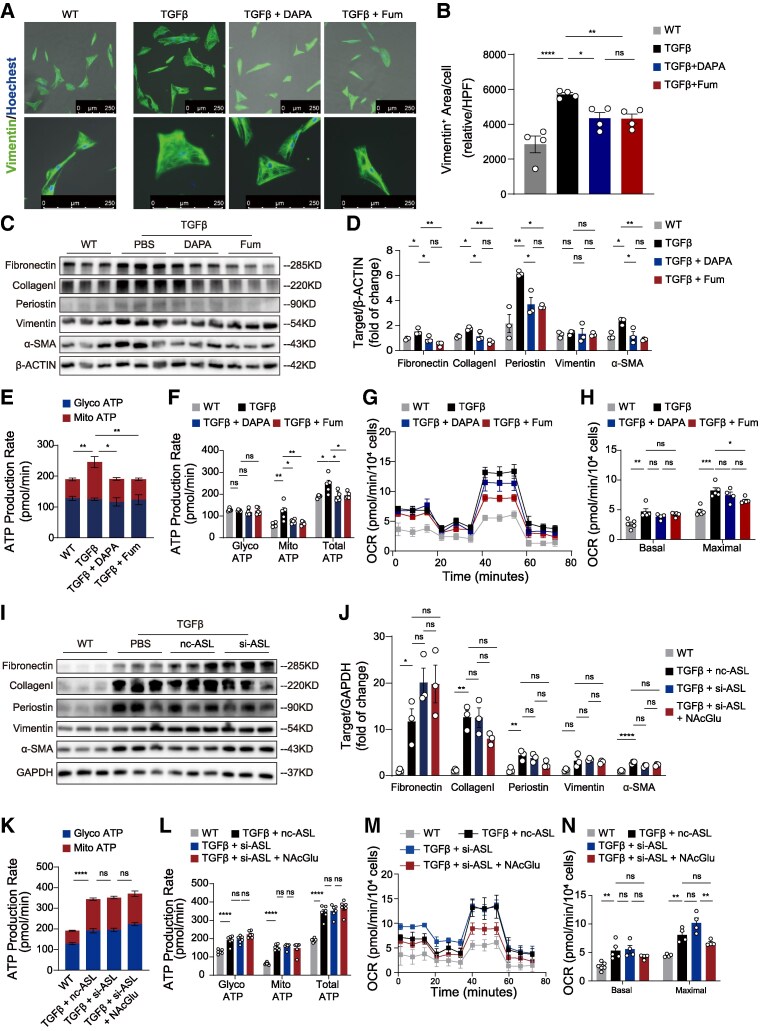
DAPA and fumarate modulate the function of HuCFs. (*A*) Immuno-fluorescence analysis of vimentin-positive HuCFs stimulated with TGF-β and treated with either DAPA or fumarate. Area per cell shown; scale bar = 250 μm. (*B*) Quantitative analysis of vimentin-positive HuCFs; *n* = 4 per group. (*C*) Western-blot analysis of fibrotic protein in HuCFs from the DMEM, TGF-β, TGF-β + DAPA, and TGF-β + Fum groups. (*D*) Quantitative analysis of (*C*); *n* = 3 per group. (*E*) ATP production rate was measured using the Seahorse XFe extracellular flux analyser. Blue indicates ECAR and red indicates OCR. The statistical results were derived from the mito ATP in each group, **P < 0.01. (*F*) Quantitative analysis of glyco ATP and mito ATP; *n* = 4 in the DMEM group, *n* = 5 in the TGF-β, TGF-β + DAPA, and TGF-β + Fum groups. (*G*) OCR in live fibroblasts was recorded and analysed using the Seahorse XFe extracellular flux analyser. (*H*) Quantitative analysis of basal and maximal respiration; *n* = 5 in all groups. (*I*) Western-blot analysis of fibrotic proteins in HuCFs from the DMEM, TGF-β, TGF-β + nc-ASL, and TGF-β + si-ASL groups. (*J*) Quantitative analysis of (*I*); *n* = 3 per group. (*K*) ATP production rate was assessed using the Seahorse XFe extracellular flux analyser. Blue represents ECAR; red represents OCR. The statistical results were derived from the mito ATP in each group, *P < 0.05, ****P < 0.0001. (*L*) Quantitative analysis of glyco ATP and mito ATP is shown. *n* = 4 in the DMEM group, *n* = 4 in each groups. (*M*) OCR of fibroblast in live cell recorded and analysed using a Seahorse XFe extracellular flux analyser. (*N*) Quantitative analysis of basal and maximal respiration was plotted. *n* = 6 in the DMEM group, *n* = 5 in TGF-β group, *n* = 4 in the TGF-β + nc-ASL group and *n* = 4 in the TGF-β + si-ASL groups. Data are presented as mean ± S.E.M. One-way ANOVA followed by Tukey's *post hoc* multiple comparisons test was used in (*B*), (*D*), (*F*), (*H*), (*J*), (*L*), and (*N*). *ns*, not significant; *P < 0.05, **P < 0.01, ***P < 0.001.

To evaluate the translational potential of our findings, we investigated whether the identified metabolic axis also operates in human cardiac fibroblasts (HuCFs). We isolated HuCFs from healthy human hearts and stimulated them with TGF-β. Strikingly, both DAPA and its downstream metabolite, fumarate, significantly attenuated the TGF-β-induced activation of HuCFs, as evidenced by reduced expression of fibrotic markers (*Figure 7A–D*). Functionally, both treatments effectively suppressed the elevated mitochondrial ATP production and OCR in human fibroblasts that was similar with that in rodent fibroblasts (*Figure 7E–H*). To genetically validate the role of this pathway in humans, we knocked down ASL (si-ASL) in HuCFs. Consistent with our model, ASL deficiency in human cells exacerbated the fibrotic response and augmented mitochondrial energy production (*Figure 7I–N*), revealing ASL's important role as a negative regulator of fibrosis across species. In addition, we collected HuCFs from patients with MI and treated them with DAPA. We observed that the expression levels of fibrosis-related proteins in HuCFs of patients with MI were decreased but without a significant difference (see Supplementary material online, Figure  *S10A* and *B*). In addition, we observed a decrease in OCR and mitochondrial ATP production without a significant difference in ECAR (see Supplementary material online, Figure  *S10C*–*H*). These results suggest that the anti-fibrotic pathway involving the NAcGlu/ASL/fumarate axis is functionally conserved in HuCFs. While the magnitude of effect on some pro-fibrotic markers was variable, the consistent trend towards their reduction, coupled with the significant suppression of mitochondrial respiration by DAPA, supports the potential therapeutic value of exploring the modulation of this metabolic pathway to mitigate cardiac fibrosis.

## 4. Discussion

We demonstrated that DAPA effectively alleviates adverse cardiac remodelling and is associated with restricted mitochondrial ATP production following MI. Our data highlight the NAcGlu/ASL/fumarate metabolic loop, which links the urea cycle with the TCA cycle, as an important component of this effect and may represent a potential target for MI treatment and prevention of pathological remodelling. Importantly, these metabolic alterations were replicated in both rodent and HuCFs. Our findings expand current knowledge of post-MI energy metabolism in cardiac fibroblasts, particularly mitochondrial function.

Previous studies have suggested that SGLT2 inhibitors enhance myocardial metabolic efficiency by promoting the generation of more energy-efficient fuels, such as ketone bodies, to support ATP production.^26–28^ This aligns with our findings that SGLT2 inhibitors mitigate adverse cardiac remodelling and functional decline by modulating ATP production. ATP acts as the cellular energy currency, enabling important biochemical processes and supporting cellular activities. Processes such as fibroblast trans-differentiation and collagen secretion are ATP-dependent,^8–11^ positioning ATP as a key regulator of fibrotic responses. Our study introduces a novel perspective: SGLT2 inhibitors can selectively attenuate mitochondrial ATP production without affecting glycolysis. In doing so, these inhibitors are associated with constrained trans-differentiation of cardiac fibroblasts, thereby linked to reduced cardiac fibrosis and improved post-MI outcomes.

We defined the process by which ammonia produced in mitochondria undergoes a series of reactions, requiring the indispensable participation of various amino acids such as ornithine to synthesize urea as the urea cycle.^29,30^ At the onset of the cycle, carbamyl phosphate reacts with ornithine within mitochondria to generate citrulline, with the essential cofactor NAcGlu facilitating this step. As NAcGlu levels increased, we observed elevated ASL activity, an enzyme that forms an important bridge between the urea and TCA cycles. In the urea cycle, ASL cleaves argininosuccinate into arginine and fumarate. Stepwise, rising NAcGlu levels lead to enhanced ASL expression and subsequent fumarate accumulation. This accumulation can inhibit key enzymes in the aerobic respiratory chain of the TCA cycle, thereby reducing mitochondrial ATP production. Consistent with findings that key metabolites and enzymes can influence fibroblast trans-differentiation,^31^ our study revealed a strong link between the urea and TCA cycles via the NAcGlu/ASL/fumarate axis.

There have been previous studies on the *N*-acetylglycine family and its association with fibrosis.^32^ Our research focused specifically on the unique metabolite NAcGlu and its role in integrating into the urea cycle through the enzyme ASL. Our research has expanded and strengthened our understanding of the *N*-acetylglycine family. We identified novel metabolic targets for modulating cellular energy metabolism, showing that NAcGlu levels influence fumarate accumulation in cardiac fibroblasts, thereby suppressing TCA cycle activity. We discovered that modulating the NAcGlu/ASL/fumarate axis can prevent HuCFs from activation and trans-differentiation in response to TGF-β stimulation. A comprehensive understanding of the complex pathogenesis such as inflammation, apoptosis and other mechanisms that trigger cardiac fibrosis after MI, is crucial for developing effective strategies to prevent heart failure. In recent years, growing research into targeted regulation of metabolic pathways for treating MI has provided a solid theoretical basis for clinical exploration of new MI treatment regimens.^33,34^ Our study expanded current knowledge of fibroblast energy metabolism following MI, offering a new perspective for alleviating cardiac fibrosis by targeting the expression or activity of key enzymes and the production of crucial intermediates bridging the urea and TCA cycles. Our results suggested that the NAcGlu/ASL/fumarate axis plays an important role in reducing cardiac fibrosis after MI. This finding raises the possibility that administering fumarate lozenges or ASL agonists could be considered for exploration as potential adjunct therapeutic options for MI patients. If supported by further research, future clinical studies would be warranted.

We noticed that a recent clinical research elucidated that DAPA treatment has not shown an obvious difference in reducing the risk of cardiovascular death or heart failure-related hospitalization compared with placebo.^35,36^ First of all, we acknowledge that animal models, while invaluable for elucidating mechanisms, have inherent limitations. Our study uses inbred mice with a standardized, severe MI, leading to a uniform and rapid disease process. In contrast, patients in the DAPA-MI trial represented a highly heterogeneous population with diverse genetic backgrounds, co-morbidities, and infarct sizes. This biological variability in humans makes it considerably more challenging to detect a uniform treatment effect. In addition, a critical distinction lies in the patient population and standard care. The DAPA-MI trial enrolled a low-risk cohort without diabetes or heart failure, and all patients received guideline-directed optimal therapy, including percutaneous coronary intervention. The annualized event rate in the placebo group was very low. In such a setting, demonstrating a statistically significant additional benefit from DAPA on top of already highly effective background care requires an immense sample size and longer follow-up. Our animal model, devoid of standard human post-MI care, represents a high-risk scenario where the absolute benefit of an effective intervention is more pronounced and easier to detect. Besides, the timing of DAPA administration is another plausible explanation. We now suggest that future research should focus on higher-risk post-MI populations where the absolute risk and potential for absolute benefit are greater. Furthermore, longer-term follow-up in both animal models and clinical trials may be needed to observe the translation of reduced fibrosis into improved clinical outcomes.

In conclusion, our research identifies a novel metabolic pathway defined by the NAcGlu/ASL/fumarate axis, which is associated with the regulation of the trans-differentiation and collagen secretion of cardiac fibroblasts by modulating mitochondrial ATP production. This mechanism is linked to the alleviation of adverse cardiac remodelling post-MI. Our findings could inform the development of a potential therapeutic strategy for cardioprotection against excessive fibrosis and long-term heart failure.

Translational perspective Our findings identified a novel potential metabolic pathway in cardiac fibroblast that regulated by SGLT2 inhibitor treatment. The urea cycle and TCA cycle are revealed to be significant steps in promoting cardiac fibroblast activation after MI. Fumarate from urea cycle is determined as a key metabolite in balancing cardiac fibroblast energy production. These findings unveil a novel potential metabolic pathway, in which relevant metabolites can be exploited as therapeutic targets or biomarkers in MI.

## Supplementary Material

cvag119_Supplementary_Data

## Data Availability

The data supporting this study’s findings are available from the corresponding author upon reasonable request.
