# Supplementary material for: Urea cycle fumarate limits fibrosis post-myocardial infarction by reducing fibroblast mitochondrial adenosine triphosphate production
Source: Cardiovasc Res. 2026 May 28;122(10):1359–73. doi: 10.1093/cvr/cvag119 (PMC13355841; doi:10.1093/cvr/cvag119)
Supplement: cvag119_Supplementary_Data [file cvag119_supplementary_data.zip › Supplementary+Materials+R2.docx]

**Urea cycle fumarate limits fibrosis post-MI by reducing fibroblast mitochondrial ATP production**

Short title: Urea cycle metabolism and cardiac fibrosis

Jing Zhao MSc^*1,2,3,4^, Yating Ruan MSc^*1,2,3,4^, Yongjian Chen PhD^5^, Qiming Chen MSc^1,2,3,4^, Tingting Hong MSc^1,2,3,4^, Linjun Wang MD, PhD^1,2,3,4^, Yinghui Xu PhD^1,2,3,4^, Liya Hou MSc^1,2,3,4^, Fei Liao PhD^1,2,3,4^, Deling Yin PhD^1,2,3,4^, Cheng Ni MD, PhD^# 1,2,3,4^

^1^Department of Cardiology of The Second Affiliated Hospital, School of Medicine, Zhejiang University, 88# Jiefang road, Hangzhou 310009, Zhejiang Province, China

^2^State Key Laboratory of Transvascular Implantation Devices,

^3^Heart Regeneration and Repair Key Laboratory of Zhejiang province,

^4^Transvascular Implantation Devices Research Institute, Binjiang Institute of Zhejiang University,

^5^Department of Cardiology, The First Affiliated Hospital of Wenzhou Medical University, Wenzhou, 325000, China

## Supplementary Methods

**Human heart samples, Isolation of Human cardiac fibroblast and ethics.**

Tissue samples were taken from the free wall of the left ventricle from two types of donated hearts: one type was derived from individuals without organic lesions or brain-dead patients (serving as the control group), and the other type was derived from patients who developed MI-related heart failure and subsequently underwent heart transplantation. All procedures were approved by the Ethics Review Committee of the Second Affiliated Hospital of Zhejiang University. All subjects were fully informed and signed a consent by patients or their relatives before sample collection. To isolate human cardiac fibroblasts (HuCFs), hearts were excised and rinsed in cold Hank’s balanced salt solution. The tissue was minced into 1 mm^3^ pieces and digested at 37°C for 15 min using type II collagenase (100 U/mL; Worthington, USA) and pancreatin (0.6 mg/mL; Sigma, USA). After each digestion, the supernatant was collected and resuspended in DMEM (Gibco, Thermo Fisher Scientific, USA) supplemented with 10% fetal bovine serum (Thermo Fisher Scientific) and 1% antibiotic solution. The digestion process was repeated (typically six times) until the digestion fluid became clear. All supernatants were pooled and centrifuged at 600 × g for 10 minutes. The resulting cell pellet was plated onto 100 mm culture dishes.

**Animal experiments and ethics statement**

All animal studies complied with the Declaration of Helsinki, and were approved by the Institutional Animal Research Committee of Zhejiang University (No. 2019-177) and were conducted in accordance with the Guide for the Care and Use of Laboratory Animals published by the U.S. National Institutes of Health.

All rats were obtained from the Zhejiang Experimental Animal Centre (Hangzhou, China). Male Sprague-Dawley (SD) rats aged 6-8 weeks were housed in a pathogen-free, temperature-controlled facility with a 12:12-h light-dark cycle. AAV9 vectors carrying the periostin promoter (*Postn*-promoter) with either NC or ASL-KD were injected into the normal LV wall under ultrasound guidance two weeks prior to MI surgery. The rats were anaesthetized with 2% isoflurane. A left parasternal incision (approximately 2 cm in length) was made along the 4^th^ intercostal space of the left chest. Subcutaneous tissue was bluntly dissected to expose the intercostal space, and intercostal muscles were carefully transected with ophthalmic scissors to avoid injury to intercostal blood vessels and nerves. A retractor was inserted to expand the surgical field, followed by a gentle incision of the parietal pleura to access the thoracic cavity. The left anterior descending coronary artery (LAD) was identified. Using a 6-0 polypropylene suture, the LAD was ligated approximately 2-3 mm below the left atrial appendage with a slipknot. The tightness of the knot was adjusted to ensure complete arterial occlusion, which was confirmed by immediate blanching of myocardial tissue in the perfused area. For the sham-operated group, the suture was passed under the LAD without ligation. After confirming hemostasis (no active bleeding in the thoracic cavity), the lung was repositioned to ensure full expansion. Intercostal muscles and the thoracic wall were sutured layer by layer with 5-0 silk suture, while subcutaneous tissue and skin were closed with continuous sutures. During the closure, air was gently squeezed out of the thoracic cavity to prevent pneumothorax.

Experiments were performed to (1) assess the central role of the SGLT2i inhibitor dapagliflozin (DAPA) (10 mg/kg) in MI and (2) determine whether ASL knockdown inhibits the therapeutic effects of DAPA post-MI. DAPA (10 mg/kg [body weight]) or NAcGlu (5g/kg [body weight]) was administered orally immediately by adding to the daily feed after MI induction. For the first objective, rats were randomly assigned to three groups (n = 15 per group): (1) Sham group, (2) MI group, and (3) MI with DAPA treatment (MI + SGLT2i group). DAPA (10 mg/kg in saline) or DMSO was administered orally immediately after MI induction. For the second objective, five groups of rats (n = 8 per group) were studied: (1) Sham group; (2) MI group with periostin promoter ASL-NC (MI + *Postn*-promoter ASL-NC); (3) MI group with periostin promoter ASL-NC and DAPA treatment (MI + *Postn*-promoter ASL-NC + SGLT2i ); (4) MI group with periostin promoter ASL knockdown (MI + *Postn*-promoter ASL-KD); (5) MI group with periostin promoter ASL knockdown and DAPA treatment (MI + *Postn*-promoter ASL-KD + SGLT2i). High-frequency echocardiography was performed at baseline (day-1), after MI but before treatment (day 3) and post-treatment (day 7, 14 and 28). On day 28, the rats were euthanised and their blood and heart tissues were collected for analysis. Adult rats were euthanized using 3% isoflurane inhalation followed by cervical dislocation for terminal tissue collection.

**Isolation, Culture, and Experimental Manipulation of Adult Rat Cardiac Fibroblasts (ARCFs)**

Male SD rats (4-6 weeks old) were euthanized using 3% isoflurane inhalation followed by cervical dislocation. Then, the hearts were excised, and the atria were dissected away to retain both left and right ventricles. The ventricles were minced into 1 mm³ fragments using fine scissors, followed by digestion in 15 mL of 2 mg/mL collagenase IV in a gentleMACS™ dissociator (Miltenyi Biotec) for 56 minutes.

The digested supernatant was collected into standard culture medium (RPMI 1640 supplemented with 20% fetal bovine serum [FBS]) and centrifuged at 400 × g for 5 minutes. The supernatant was discarded carefully to avoid disturbing the pellet, and 2 mL of red blood cell (RBC) lysis buffer was added to the pellet for 1 minute. This was neutralized by the addition of 4 mL of 2% bovine serum albumin (BSA) in phosphate-buffered saline (PBS), and the mixture was centrifuged again at 400 × g for 5 minutes.

After discarding the supernatant, the pellet was resuspended in 1 mL of 2% BSA/PBS and transferred to a 1.5 mL microcentrifuge tube. The cell suspension was incubated with 20 μL of purified mouse anti-rat CD32 antibody (BD Pharmingen™, 550270) for 10 minutes, followed by incubation with fluorescein isothiocyanate (FITC)-conjugated anti-rat CD45 (Biolegend, 202205, 5 μL) and phycoerythrin (PE)-conjugated anti-rat CD31 (BD Pharmingen™, 555027, 5 μL) antibodies for a further 10 minutes. ARCFs were then isolated via flow cytometry using a BD FACSAria™ Fusion cell sorter.

Sorted cells were seeded in standard culture medium (Dulbecco’s Modified Eagle’s Medium [DMEM] supplemented with 10% FBS and 1% penicillin/streptomycin) at a density of 5 × 10⁵ cells/cm² and incubated for 24 hours. The medium was subsequently replaced, and cells were cultured until reaching 80% confluence before passage. Primary ARCFs were passaged twice, and second-passage cells were used for all experimental assays. Previous studies have demonstrated that cardiac fibroblasts retain their phenotypic stability up to the 3rd passage; beyond this, spontaneous conversion to cardiac myofibroblasts occurs. Notably, ARCFs were not subjected to serum starvation, as this treatment induces cellular stress that may confound experimental results. Prior to protein analysis, cells were cultured to 80% confluence and then harvested.

Confirmation of Cell Purity

Cell purity was verified via immunofluorescence staining using the following primary antibodies: anti-vimentin (Abcam, ab92547; 1:400 dilution), anti-CD31 (Abcam, ab222783; 1:400 dilution), and anti-troponin I (Abcam, ab56357; 1:400 dilution). Staining results indicated that >95% of cultured cells were vimentin-positive, confirming high fibroblast purity. No significant contamination by endothelial cells (CD31-positive) or cardiomyocytes (troponin I-positive) was detected.

Second-passage ARCFs were seeded in 6-well plates and subjected to serum starvation to mimic the stressful microenvironment associated with myocardial infarction (MI).

**Isolation and Culture of Neonatal Rat Cardiac fibroblast (NRCFs) and Cardiomyocytes (NRCMs)**

Neonatal rat cardiac fibroblasts were isolated using the Neonatal Heart Dissociation Kit for mouse and rat (130-098-373, Miltenyi, Germany). Briefly, The neonatal rats were euthanized using 3% isoflurane inhalation followed by decapitation. Then, the hearts were collected into a 10 cm dish containing cold PBS, minced into 1–2 mm³ pieces and incubated in enzyme mix buffer at 37°C for 15 min. This digestion step was repeated twice. After digestion, the cell suspension was neutralised with culture medium containing FBS, filtered and centrifuged at 600 × g for 5 min.

NRCMs were collected from the supernatant after differential adhesion. Cells were seeded in 6-well plates (3 × 10⁵ cells/cm²) or 24-well plates (5 × 10⁴ cells/cm²) in appropriate culture medium.

**Hemodynamics**

Cardiac hemodynamic parameters were measured immediately following the final echocardiographic evaluation. In summary, adult rats were anaesthetized using 2% isoflurane inhalation. Under meticulous dissection facilitated by a stereomicroscope (SZX7, Olympus), the right common carotid artery was exposed and cannulated with a 1.4-French Millar catheter equipped with a micro-tip pressure sensor (SPR-671NR, Millar Instruments, USA). The pressure transducer was interfaced with a data acquisition system (PowerLab, AD Instruments) to continuously monitor and record key cardiovascular indices, including heart rate, arterial blood pressure, intraventricular pressures, and the maximal rates of ventricular pressure rise and fall (±dp/dt). Baseline zero reference was obtained by placing the sensor in normal saline before insertion. At the end, rats were euthanized using 3% isoflurane inhalation followed by cervical dislocation.

**Picro-sirius red staining and fibrosis calculation of rat heart**

Hearts were harvested from anesthetized rats, embedded in 4% paraffin, and sectioned into 4 μm-thick slices from the mid-papillary region to the apex at 300 μm intervals. The sections were stained with Sirius red (Sbjbio, Nanjing, China) for 30 min, followed by sequential washes with 95% and 100% ethanol. Images were captured using slide scanning system (SQS-12P, Shengqiang Technology Co., Ltd, China). To evaluate the infarct area, the mean endocardial and epicardial lengths of the fibrotic region (representing the infarct zone) were measured and expressed as a percentage of the total mean endocardial and epicardial lengths of the left ventricle. These measurements were performed using Imaging Pro software. Interstitial fibrosis was assessed by calculating the ratio of the red-stained collagen area to the total tissue area within a single field of view. Perivascular fibrosis was evaluated by measuring the collagen-stained (red) area relative to the total vessel area, where the outer edge of the fibrotic region marked the boundary of the entire vessel. All image analyses were performed using Image Pro Plus software version 6.0.

**Immuno-fluorescence staining**
For immuno-fluorescence, rat hearts at 3 days and 7 days post-MI were dehydrated in 30% sucrose solution, embedded in Tissue-Tek OCT compound, snap-frozen in dry ice, and then cut into 7 μm sections. The sections were then stained with In Situ Cell Death Detection Kit (11684817910, MERCK, USA), CD3 (ab16669, Abcam, United Kingdom), CD68 (ab125212, Abcam, United Kingdom), Cardiac Troponin I (ab188877, Abcam, United Kingdom), and DAPI (Vector Laboratories, Burlingame, CA, United States). For intracellular protein detection, cells were fixed with 4% paraformaldehyde at room temperature for 10 minutes, followed by permeabilization with 0.1% Triton X-100 for 10 minutes at room temperature. Non-specific binding sites were blocked with 5% BSA/PBS for 60 minutes, and cells were then incubated overnight at 4°C with primary antibodies: anti-Ki67 (Abcam, ab16667; 1:200 dilution), anti-vimentin (Abcam, ab20346; 1:200 dilution), anti-CD31 (Abcam, ab222783; 1:100 dilution), and anti-troponin I (Abcam, ab56357; 1:400 dilution).

After washing with PBS, cells were incubated with secondary antibodies conjugated to Alexa Fluor® 488 (Invitrogen, A32814TR; 1:400 dilution), Alexa Fluor® 555 (Invitrogen, A32773; 1:500 dilution), or Alexa Fluor® 647 (Invitrogen, A32795TR; 1:500 dilution) for 1 hour at room temperature in 1% BSA/PBS. Nuclei were counterstained with 4',6-diamidino-2-phenylindole (DAPI). Immunofluorescence images were acquired using a TissueFAXS Cytometry Fluorescence Scanner and analyzed with StrataQuest software (v8.0.70).

Rat hearts at 28 days post-MI were fixed in 10% formalin-PBS, then embedded in paraffin, and cut into 4 μm sections. After deparaffinization, rehydration and tissue antigen recovery, the sections were stained with WGA-Alexa Fluor 594 (W11262, Thermo Fisher, USA), Cardiac Troponin I (ab47003, Abcam, United Kingdom), IB4 (I32450, MERCK, USA) and DAPI (Vector Laboratories, Burlingame, CA, USA).

**Ultrasound-guided intramyocardial injection**

14 days post-MI rats were subjected to 1.5-2.5% isoflurane anesthesia with 1 L/min 100% oxygen. Rats were placed supine on a platform (Vevo3100, Visual Sonics), and allowed the heart (in the left side of the chest) to face the syringe clamp and needle. Use the animal platform adjustment controls to adjust the field of view and to target any desired injection site in the left ventricular myocardium. Then the intramyocardial injection was performed via 30 G needle with 1 mL syringe. Three injections of postn-AAV9-shASL (3.3E+11 v.g./mL) diluted in PBS to a total volume of 10 μL or 10 μL Scramble was slowly injected into three different sites of anterior wall of the left ventricle in a controlled manner by a blinded technician. A transient echobright appearance to the injected myocardial region may hint the successful injection.

**Quasi-targeted metabolomics**

NRCFs were seeded in 6 cm dishes at a density of 2 ×106 cells and cultured to 80% confluence. Cells were then treated with TGFβ (10 ng/mL) and/or dapagliflozin (10 μM) for 24 h. After treatment, cells were freeze-dried and resuspended in prechilled 80% methanol, followed by thorough vortexing. Samples were incubated on ice for 5 min and subjected to three cycles of liquid nitrogen lysis. Lysates were centrifuged at 15,000 × g, 4°C for 15 min. A portion of the supernatant was diluted with LC-MS grade water to a final concentration of 80% methanol. Samples were transferred to fresh Eppendorf tubes and centrifuged again at 15,000 × g, 4°C for 15 min. Finally, the supernatant was used for LC-MS/MS system analysis.

**^13^C-Labelled targeted metabolic flux analysis**

MS measurement of isotopologue distribution was performed using a Thermo Q Exactive Plus hybrid quadrupole Orbitrap mass spectrometer coupled to a Thermo Vanquish UPLC system. Instrument performance optimisation and routine maintenance were conducted every 48 h. Phase separation yielded an aqueous upper phase containing polar metabolites and an organic lower phase containing nonpolar metabolites. The aqueous phase was transferred to microcentrifuge tubes, while the lower chloroform phase was collected in glass tubes. For reverse-phase liquid chromatography separation, an ACQUITY UPLC BEH C18 column (100 × 2.1 mm, 1.7 μm; Waters) was used. Mass spectrometric data were acquired using negative ion electrospray ionisation on the Q Exactive Plus system. The scan range was set from 50 to 1000 m/z, with a scan time of 0.2 s per function. Ion monitoring parameters included a capillary voltage of 2.0 kV, a source temperature of 120°C and a desolvation temperature of 500°C. Data processing and ion annotation, based on accurate mass, was performed using TraceFinder 5.0 and Xcalibur 4.0 (Thermo). Metabolite mass isotopomer distributions were determined by calculating the ratio of each isotopomer’s integrated peak area to the total integrated peak areas of all possible isotopomers for the respective metabolite.

**Western blot**

Proteins were extracted from apex heart tissue 7 days and 28 days post-MI and lysed using RIPA buffer supplemented with protease and phosphatase inhibitor cocktails (WD316307, Thermo Fisher Scientific, USA). Cells were washed twice with PBS, then lysed using RIPA buffer supplemented with protease and phosphatase inhibitor cocktails (WD316307, Thermo Fisher Scientific, USA). Protein concentrations were quantified using the BCA Protein Assay Kit (Thermo Fisher Scientific, MA).

Sodium dodecyl sulfate-polyacrylamide gel electrophoresis (SDS-PAGE) was performed according to standard protocols to separate proteins, which were then transferred to PVDF membranes. The details of the antibodies used are provided in Supplementary Table 2.

**ARCF Migration Assay**

ARCFs were seeded in 6-well plates at a density of 5 × 10⁵ cells/cm² and cultured until reaching 80% confluence on day 2. A straight, cell-free zone was created across the cell monolayer in each well using a 100 μL micropipette tip to simulate an in vitro wound. Following wound healing, the monolayer was washed with basal medium to remove cell debris, and complete medium was added. Cells were cultured at 37°C in a 5% CO₂ incubator. Wound width was imaged using a Leica DMIL bright-field microscope on day 1 and day 2 post-wounding to assess cell migration.

**TGF-β stimulation**

Cells were incubated in serum-free medium (DMEM supplemented with 1% penicillin/streptomycin) for 24 hours in a standard cell incubator. Following overnight starvation, 10 ng/mL transforming growth factor-β (TGF-β; Perptech, 100-21C) was added to fresh standard medium (DMEM + 1% penicillin/streptomycin), and cells were incubated for an additional 24 hours.

**siRNA transfection**

NRCFs were seeded in plates and cultured to 80% confluence, then transfected with N-acetyl-glutamate synthase (NAGS) siRNA, ASL siRNA or control siRNA using Lipofectamine RNAiMAX transfection reagent (13778, Thermo Fisher Scientific, MA) for 24 h. The siRNA sequences are listed Supplementary Table 3.

**Enzymes site mutation**

A full-length NAGS plasmid containing C394, C437 and C467–472 mutations (plasmid-mut-NAGS) was constructed by GenePharma (Shanghai). NRCFs were cultured to 80% confluence and transfected with X-tremeGENE HP DNA Transfection Reagent (06366236001, Merck, USA) for 48 h.

**Enzyme activity and fumarate assay measurements**

The enzyme activities of IDH, CS and α-KGDH, as well as the fumarate content in NRCFs, were measured according to the manufacturer’s instructions for the respective assay kits. Blood was collected from sacrificed rats, and serum was immediately separated by centrifugation at 4,000 × g for 10 min at 4°C. The serum was stored at 80°C until further analysis.

**Metabolic Assay**

ARCFs sorted via flow cytometry from the Sham, MI, and MI + DAPA groups were seeded at a density of >1 × 10⁶ cells per sample. Cells were collected into centrifuge tubes, the supernatant was discarded, and residual liquid was aspirated. Cells were lysed with 100 μL BeyoLysis™ buffer, and the concentrations of fumarate (Beyotime, S0517) and malate (Beyotime, S0511) were detected using standard samples according to the manufacturer’s protocols.

**ELISA Assay**

The concentrations of tumour necrosis factor-α (TNF-α; Mlbio, cat. no. ml002859), interferon-γ (IFNγ; Mlbio, cat. no. ml0064291), interleukin-6 (IL-6; Mlbio, cat. no. ml0064292), interleukin-1β (IL-1β; Mlbio, cat. no. ml037373), interleukin-2 (IL-2; Mlbio, cat. no. ml107051), and interleukin-10 (IL-10; Mlbio, cat. no. ml037371) in apex tissue and serum were measured 7 days post-MI using commercial ELISA kits, following the manufacturer’s instructions. Briefly, 20 mg of apex tissue was homogenized in 180 μL PBS supplemented with protease and phosphatase inhibitors using a tissue grinder.

**NRCM Apoptosis Assay**

NRCMs were subjected to serum and glucose starvation by incubation in serum- and glucose-free DMEM supplemented with 1% penicillin/streptomycin. Cells were then placed in a 1% O₂ hypoxic incubator (Whitley H35 Hypoxystation) for 24 hours. Following hypoxic treatment, cells were collected and stained with 5 μL fluorescein isothiocyanate (FITC)-conjugated Annexin V for 15 minutes and propidium iodide (PI) for 10 minutes in the dark. The stained cell suspension was diluted with 300 μL of binding buffer, and apoptosis was analyzed via flow cytometry using a BD Fortessa™ cell analyzer.

**NRCM TUNEL Assay**

NRCMs were treated as described in the apoptosis assay. Cells were fixed with 4% paraformaldehyde at room temperature for 10 minutes, permeabilized with 0.1% Triton X-100 for 10 minutes, and blocked with 5% BSA/PBS for 60 minutes. Cells were incubated overnight at 4°C with anti-troponin I primary antibody (Abcam, ab56357; 1:400 dilution), followed by incubation with Alexa Fluor® 488-conjugated secondary antibody (Invitrogen, A32814TR; 1:400 dilution) for 1 hour at room temperature.

Subsequently, cells were incubated with proteinase K (20 μg/mL) at 37°C for 20 minutes. After three washes with PBS, terminal deoxynucleotidyl transferase (TdT) enzyme and Cy3-labeled dUTP mixture (Beyotime, TUNEL Assay Kit, cat. no. C1090) were added, and cells were incubated at 37°C in the dark for 1 hour. TUNEL-stained cells were imaged using a TissueFAXS Cytometry Fluorescence Scanner and analyzed with StrataQuest software (v8.0.70).

#### Abbreviations and Acronyms

| **AMI** | Acute Myocardial Infarction |
| --- | --- |
| **SGLT2i** | Sodium-glucose cotransporter2 inhibitor, SGLT2i |
| **LV** | Left ventricular |
| **LVPW;d** | Left ventricular posterior wall, diastolic |
| **LVPW;s** | Left ventricular posterior wall, systolic |
| **LVID;d** | Left ventricular internal diameter, diastolic |
| **LVID;s** | Left ventricular internal diameter, systolic |
| **EF** | Ejection fraction |
| **FS** | Fractional shortening |
| **ARCF** | Adult rat cardiac fibroblast |
| **HuCF** | Human cardiac fibroblast |
| **NRCF** | Neonatal rat cardiac fibroblast |
| **WT** | Wild-type |
| **TGF-β** | Transforming growth factor beta |
| **WGA** | Wheat Germ Agglutinin |
| **cTNI** | Cardiac Troponin I |
| **IDH** | Isocitrate dehydrogenase |
| **CS** | Citrate synthase |
| **α-KGDH** | 2-Oxoglutarate dehydrogenase |
| **DAPA** | Dapagliflozin |
| **NAGS** | N-acetylglutamate synthase |
| **ASL** | Argininosuccinate Lyase |
| **NAcGlu** | N-acetyl-glutamic acid |
| **CarGlu** | Carglumic acid |
| **Fum** | fumarate |
| **AAV9** | adeno-associated virus 9 |

**Antibodies**

For Flow cytometry and cell sorting, the following antibodies were used: CD45-FITC (Biolegend, 202205, 1:200 dilution), CD31-PE (BD Pharmingen™, 555027, 1:200 dilution),

For immunofluorescence staining, the primary and secondary antibodies used in this study included Vimentin (Abcam, ab92547; 1:400 dilution), CD31 (Abcam, ab222783; 1:400 dilution), CD3(Abcam, ab16669; 1:400 dilution), CD68 (Abcam, ab125212; 1:400 dilution), Ki67 (Abcam, ab16667; 1:200 dilution), vimentin (Abcam, ab20346; 1:200 dilution), Cardiac Troponin I (Abcam, ab56357; 1:400 dilution), secondary antibodies conjugated to Alexa Fluor® 488 (Invitrogen, A32814TR; 1:400 dilution), Alexa Fluor® 555 (Invitrogen, A32773; 1:500 dilution), or Alexa Fluor® 647 (Invitrogen, A32795TR; 1:500 dilution).

For western-blot analysis, the primary antibodies used in this study included anti-Fibronectin (Abcam, ab2413; 1:1,000 dilution), anti-Collagen I (Abcam, ab270993; 1:1,000 dilution), anti-Collagen I (Abcam, ab34710; 1:1,000 dilution), anti-Periostin (R&D, AF2955; 1:500 dilution), anti-Periostin (Abcam, ab14041; 1:1,000 dilution), anti-α-SMA (Abcam, ab5694; 1:5,000 dilution), anti-ASL (Abcam, ab201026; 1:1,000 dilution),anti-NAGS (FineTest, FNab05539; 1:1,000 dilution), p-P38 (CST, cat. no. 4092; 1:1000 dilution), P38 (CST, cat. no. 9212; 1:1,000 dilution), p-ERK(CST, cat. no. 4370; 1:1000 dilution), ERK (CST, cat. no. 4695; 1:1000 dilution), p-GSK3β (CST, cat. no. 5558; 1:1000 dilution), GSK3β (CST, cat. no. 12456; 1:1000 dilution), p-AKT (CST, cat. no. 4060; 1:1000 dilution), AKT (Abcam, ab8805; 1:1000 dilution), p-STAT3 (CST, cat. no. 9134; 1:1000 dilution), STAT3 (CST, cat. no. 4904; 1:1000 dilution), GAPDH (CST, cat. no. 2118; 1:5,000 dilution) and β-actin (CST, cat. no. 4967; 1:5,000 dilution), and HRP-conjugated secondary antibodies (CST, cat. no. 7074/7076/7077; 1:5,000 dilution).

**Supplementary Figure Legends**

**Supplementary Figure 1**


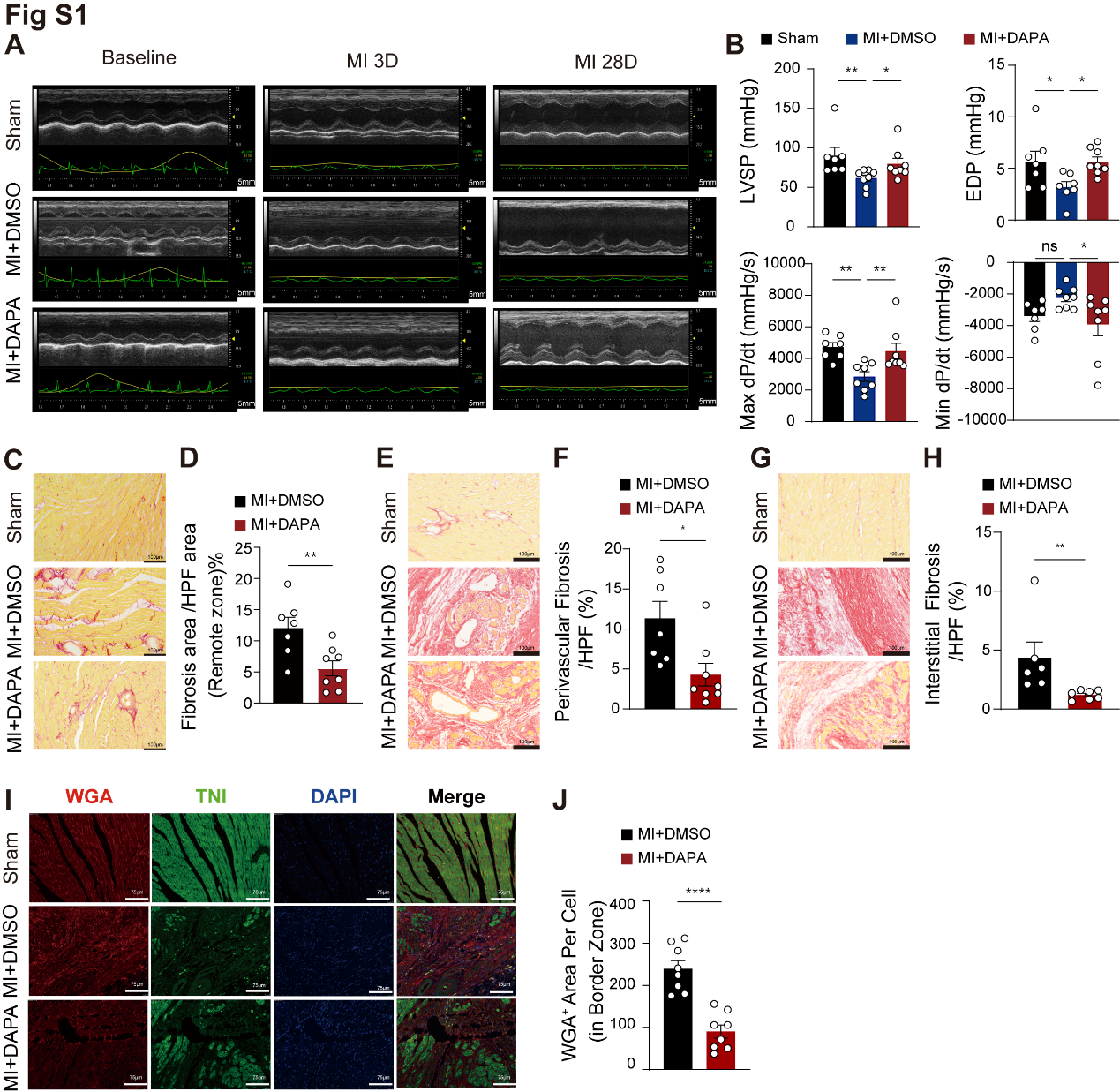


**Fig. S1. DAPA treatment in MI model.**

(A) Representative images of M-mode echocardiography in each group at different time points. Scale bar = 2 mm. (B) Quantitative analysis of hemodynamics. *n =* 7-8 in each group. *ns*, not significant; *p<0.05, **p<0.01. (C) Representative images of Picro-Sirius red staining from heart sections at remote zone on rat from each experimental group. Scale bar = 100 μm (D) Summary data on fibrosis area; *n =* 7 in MI+DMSO group, *n =* 8 in MI+DAPA group. **p<0.01. (E) Representative images of Picro-Sirius red staining from perivascular fibrosis at infarct zone on rat from each experimental group. Scale bar = 100 μm. (F) Summary data on fibrosis area; *n =* 7 in MI+DMSO group, *n =* 8 in MI+DAPA group. **p*<0.05. (G) Representative images of Picro-Sirius red staining from interstitial fibrosis at infarct zone on rat from each experimental group. Scale bar = 100 μm. (H) Summary data on fibrosis area; *n =* 6 in MI+DMSO group, *n =* 7 in MI+DAPA group. ***p*<0.01. (I) Representative images of WGA immunostaining images from heart sections on rat from each experimental group. Scale bar = 75 μm. (J) Summary data on the proportion of WGA-positive area in each border zone; *n =* 8 in MI+DMSO group and *n =* 8 in MI+DAPA group. *****p* < 0.0001. Among all statistical plots, the data are presented as the mean ± SEM. Two-tailed Student’s t-test was employed to compare two independent groups conducted in (D), (F), (H). One-way ANOVA followed by Tukey’s post hoc multiple comparisons test was conducted in (B) and (J). *ns*, not significant; **p*<0.05, ***p*<0.01, ****p*<0.001, *****p* < 0.0001.

**Supplementary Figure 2**

**
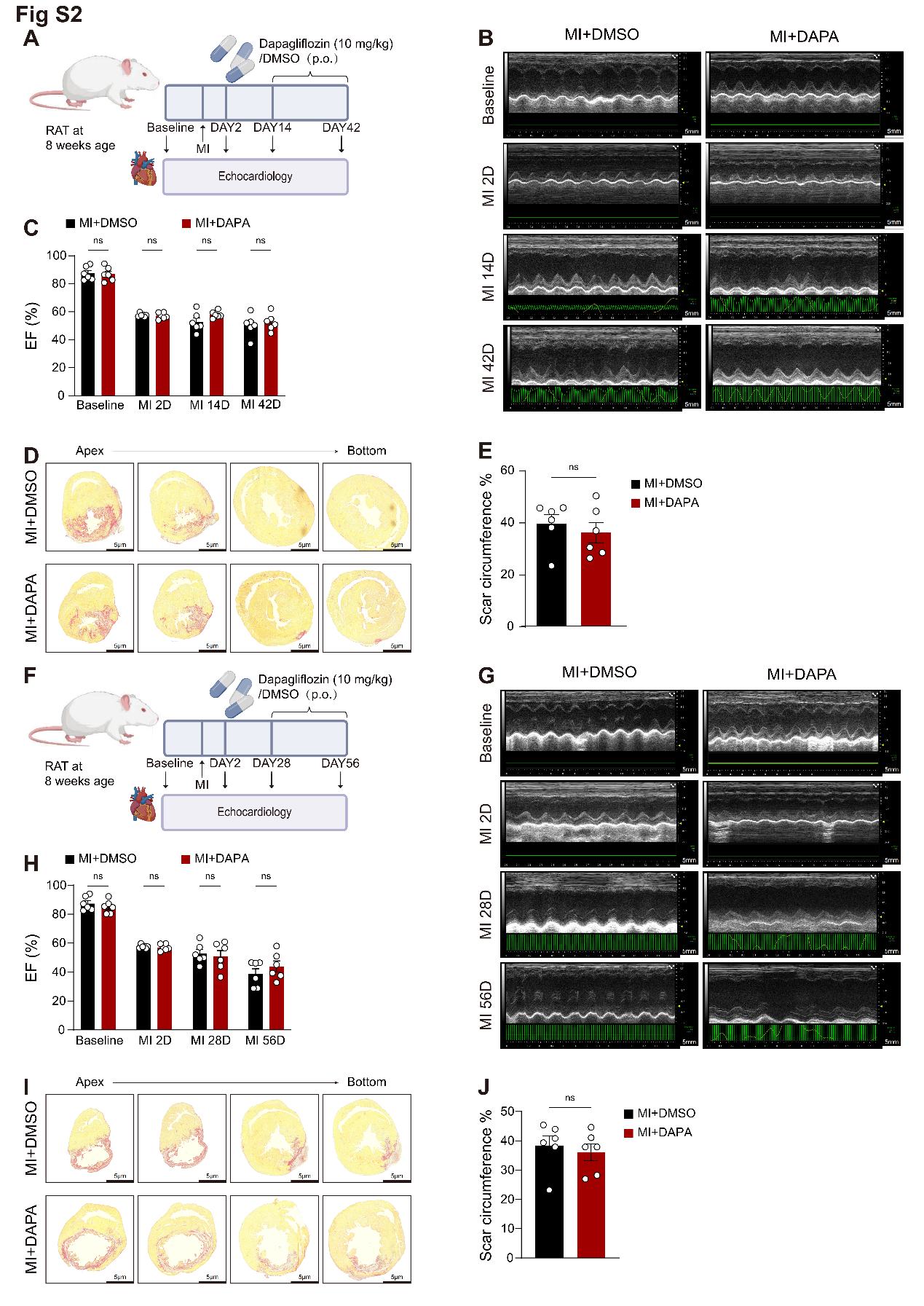
Fig. S2. The role of DAPA administration at the late stage of MI on cardiac function and fibrosis in rats.**

**(**A) Schematic representation of the experimental design for DAPA treatment starting at 14 days after myocardial infarction in rats. (B) Representative images of M-mode echocardiography in each group at different time points. Scale bar = 5 mm. Administration of DAPA at the post-MI 14D. (C) Quantitative echocardiographic analysis of EF; *n =* 6 per group. *ns*, not significant; (D) Representative images of Picro-Sirius red-stained heart sections from each experimental group. Scale bar = 5 mm. (E) Quantification of scar circumference ratio based on Picro-Sirius red staining; *n =* 6 per group. *ns*, not significant. (F) Schematic representation of the experimental design for DAPA treatment starting at 14 days after myocardial infarction in rats. (G) Representative images of M-mode echocardiography in each group at different time points. Scale bar = 5 mm. Administration of DAPA at the post-MI 28D. (H) Quantitative echocardiographic analysis of EF; *n =* 6 per group. *ns*, not significant; (I) Representative images of Picro-Sirius red-stained heart sections from each experimental group. Scale bar = 5 mm. (J) Quantification of scar circumference ratio based on Picro-Sirius red staining; *n =* 6 per group. *ns*, not significant. Among all statistical plots, the data are presented as the mean ± SEM. Two-way ANOVA followed by Tukey’s post-hoc test was used for statistical analysis in (C) and (H). Two-tailed Student’s t-test was employed to compare two independent groups conducted in (E) and (J). *ns*, not significant.

**Supplementary Figure 3**


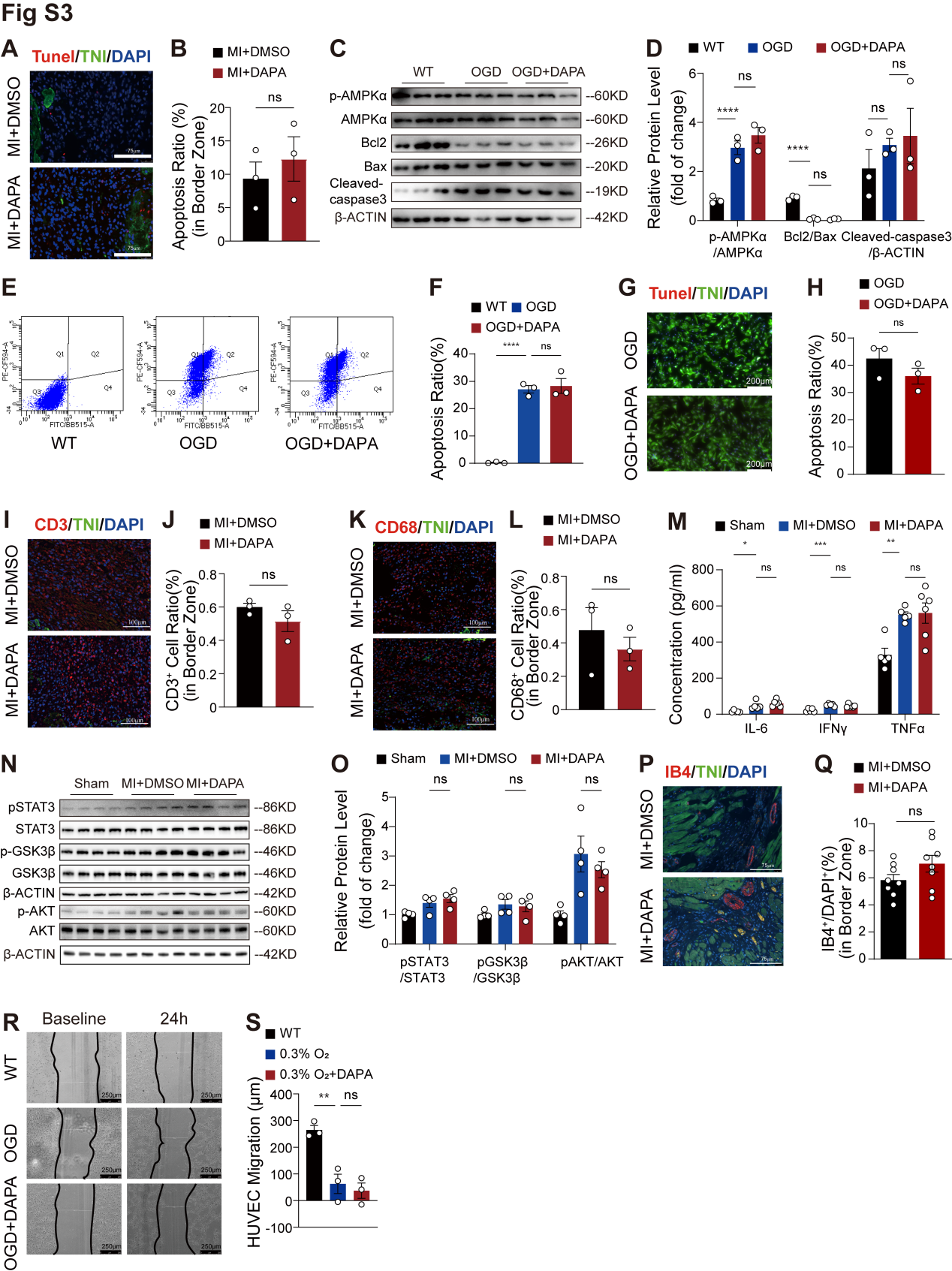


**Fig. S3. The effect of DAPA on myocardial apoptosis, inflammation, and angiogenesis in a rat model of MI**.

(A) Representative images of Tunel staining images from heart sections on rat from each experimental group, at 3 days post-MI, in which apoptotic cells were marked by Tunel (red) and cardiomyocytes were marked by Troponin I (green). Scale bar = 75 μm. (B) Summary data on the proportion of Tunel positive area in each border zone, *n =* 3 in each group. *ns*, not significant. (C) Apoptosis related protein expressions including Bcl2, Bax, Cleaved-Caspase3 and cell proliferation signal pathway protein p-AMPKα (Thr172) and AMPKα were evaluated in WT NRVMs and OGD treatment NRVMs that either received PBS or DAPA treatment for 24h. (D) Quantitative analysis of apoptosis related proteins and cell proliferation related protein are shown; *n =* 3 in each group. *ns*, not significant; *****p* < 0.0001. (E) Representative images of apoptosis assay was evaluated with FITC-Annexin V and PI staining by flow cytometry. (F) Quantitative analysis of apoptosis ratio (%), *n =* 3 in each group. *ns*, not significant; *****p* < 0.0001. (G) Representative images of TUNEL staining was evaluated by TissueFAXS Cytometry Fluorescence Scanner. Scale bar = 200 μm. (H) Quantitative analysis of apoptosis ratio (%) used by StrataQuest software (v8.0.70), *n =* 3 in each group. *ns*, not significant. (I, K) Representative images of CD3 (I) and CD68 (K) staining images from heart sections on rat from each experimental group, at 7 days post-myocardial infarction, in which T cells were marked by CD3 (red), macrophages were marked by CD68 (red) and cardiomyocytes (green) were marked by Troponin I meanwhile nuclei were marked by DAPI (blue). Scale bar = 100 μm. (J, L). Summary data on the proportion of CD3 (J) and CD68 (L) positive area in each border zone. *n =* 3 in each group. *ns*, not significant. (M) Inflammatory cytokines evaluated by Elisa kit in rat heart from Sham, MI+DMSO, MI+DAPA group, *n=5* in Sham, MI+DMSO, *n=6* in MI+DAPA group. *ns*, not significant; *p < 0.05, **p < 0.01, ****p < 0.0001. (N) Pro-inflammatory cytokine related signal pathway protein p-GSK3β(Ser9), GSK3β, p-AKT(Ser473), AKT, p-STAT3(Ser727) and STAT3 were evaluated in Sham, MI+DMSO, and MI+DAPA group. (O) Quantitative analysis of apoptosis related proteins and cell proliferation related protein are shown; *n =* 4 in each group. *ns*, not significant. (P) Representative images of IB4 staining images from heart sections on rat from each experimental group, at 28 days post-myocardial infarction in which endothelial cells were marked by IB4 (red), and cardiomyocytes (green) were marked by Troponin I (green), and nuclei were marked by DAPI (blue). Scale bar = 100 μm. (Q) Summary data on the proportion of IB4-positive area in each border zone. *n =* 8 in each group. *ns*, not significant. (R) Representative images of HUVEC in migration from WT endothelial cells and OGD-treated endothelial cells that either received PBS or DAPA treatment for 24h. Scale bar = 250 μm. (S) Summary data on the proportion of HUVEC in migration in vitro. *n =* 3 in each group. *ns*, not significant; ***p*<0.01. Among all statistical plots, the data are presented as the mean ± SEM. Two-tailed Student’s t-test was employed to compare two independent groups conducted in (B), (F), (H), (J), (L), (Q) and (S), One-way ANOVA followed by Tukey’s post hoc multiple comparisons test was conducted in (D), (M) and (O), *ns*, not significant; **p*<0.05, ***p*<0.01, ****p*<0.001, *****p* < 0.0001.

**Supplementary Figure 4**


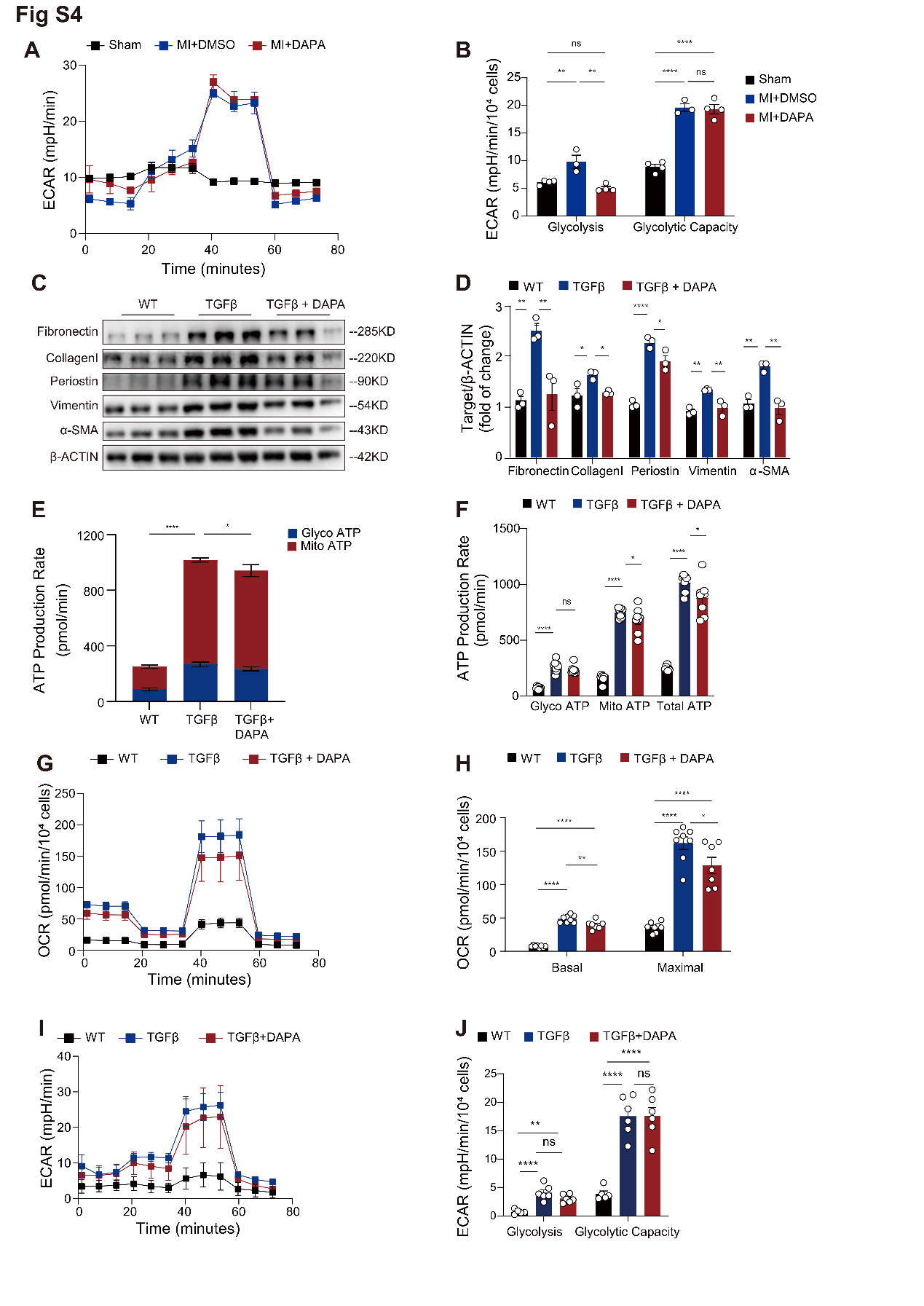


**Fig. S4. DAPA attenuates cardiac fibrosis and reduces fibroblast mitochondrial ATP production in neonatal and adult derived rat cardiac fibroblasts.**

(A) The extracellular acidification rate (ECAR) of ARCFs which were isolated from Sham, MI+DMSO and MI+DAPA group was recorded and analyzed using Seahorse XFe extracellular flux analyzer. (B) Quantitative analysis of glycolysis and glycolytic capacity was plotted. *n =* 3-4 in each group. *ns*, not significant; ***p* < 0.01, *****p* < 0.0001. (C) Detection of fibrotic proteins in WT NRCFs, with TGF-β alone or in combination with DAPA. (D) Quantitative analysis of fibrotic protein expression; *n =* 3 per group. (E) ATP production rate measured using a Seahorse XFe extracellular flux analyser. The statistical results were derived from the mito ATP in each group, **p* < 0.05, *****p* < 0.0001. (F) Quantification of ATP production derived from glyco ATP and mito ATP; *n =* 7-8 per group. *p < 0.05, *****p* < 0.0001. (G) The oxygen consumption rate (OCR) of NRCFs was measured by a Seahorse XFe extracellular flux analyser. (H) Quantitative analysis of basal and maximal respiration: *n =* 7-8 per group. **p* < 0.05, ***p* < 0.01, *****p* < 0.0001. (I) The ECAR of NRCFs was measured by a Seahorse XFe extracellular flux analyser. (J) Quantitative analysis of glycolysis and glycolytic capacity was plotted. *n =* 5-6 in each group. *ns*, not significant; ***p* < 0.01, *****p* < 0.0001. Among all statistical plots, the data are presented as the mean ± SEM. One-way ANOVA followed by Tukey’s post hoc multiple comparisons test was conducted in (B), (D), (E), (F), (H) and (J), *ns*, not significant; **p*<0.05, ***p*<0.01, ****p*<0.001, *****p* < 0.0001.

**Supplementary Figure 5**


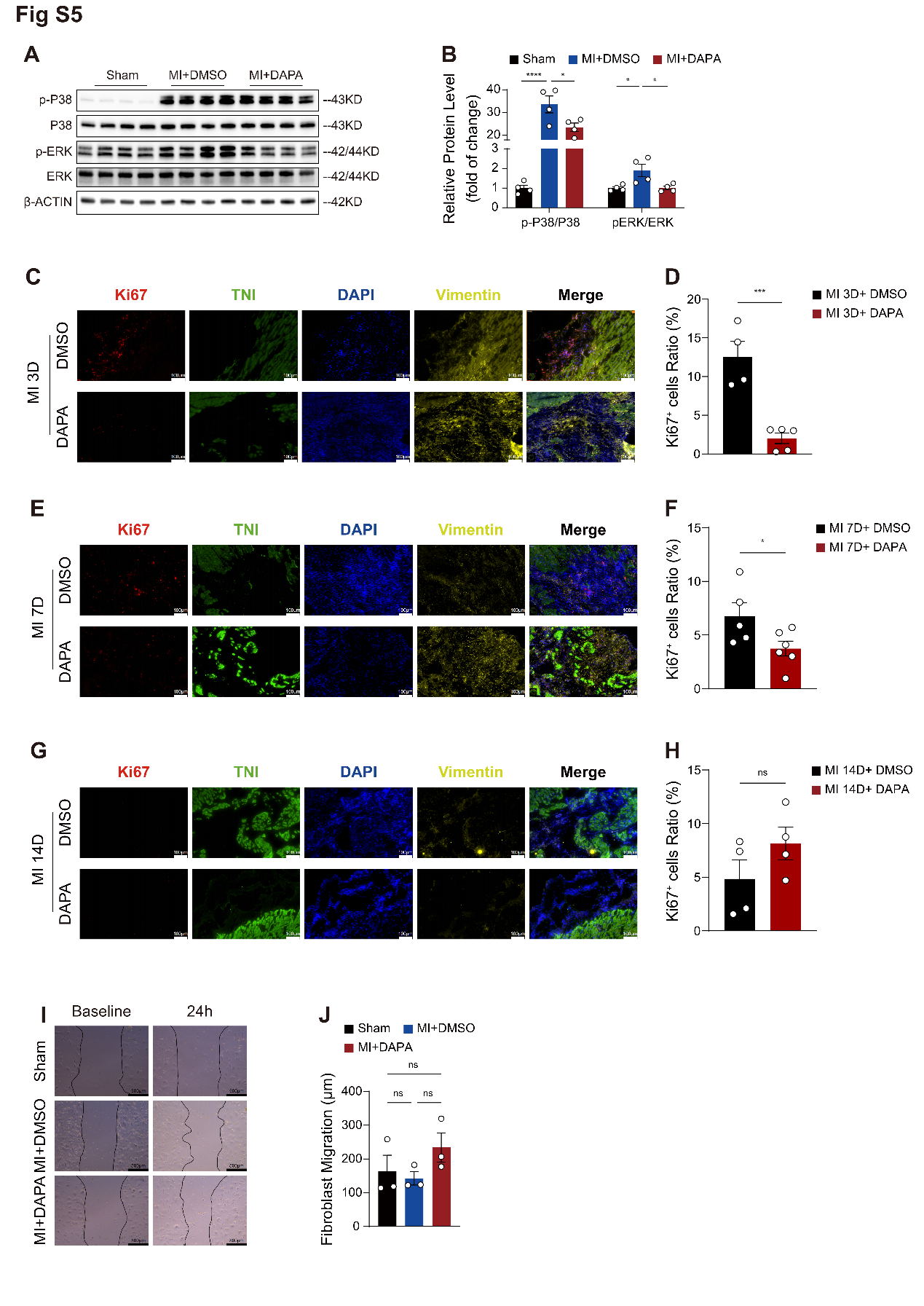


**Fig. S5. DAPA reduces cell proliferation and proliferation-related signaling pathways in a rat model of MI but does not affect cell migration.**

(A) Cell proliferation signal pathway protein p-p38MAPK(Thr180/Tyr182), p38MAPK, p-p44/42 MAPK(Erk1/2) (Thr202/Tyr204), p44/42 MAPK(Erk1/2) were evaluated in Sham, MI+DMSO and MI+DAPA group at post MI 7D. (B) Quantitative analysis of cell proliferation related protein are shown; *n =* 4 in each group. **p* < 0.05, *****p* < 0.0001. (C-H) Representative images of Ki67 immunostaining images from heart sections on rat from each experimental group at 3 days post-MI (C), 7 days post-MI (E), 14 days post-MI (G), in which proliferating cells were marked by Ki67 (red), cardiomyocytes were marked by Troponin I (green), and fibrosis were marked by Vimentin (yellow) respectively. Scale bar = 100 μm. Summary data on the proportion of Ki67 positive cell ratio at 3 days post-MI (D), 7 days post-MI (F) and 14 days post-MI (H) in each border zone. *n* = 4-6 in each group. *ns*, not significant. **p*<0.05, ****p*<0.001. (I-J) Representative images and summary data on the proportion of ARCFs which were isolated from Sham, MI+DMSO and MI+DAPA groups in migration in vitro. *n =* 3 in each group. *ns*, not significant. Among all statistical plots, the data are presented as the mean ± SEM. One-way ANOVA followed by Tukey’s post hoc multiple comparisons test was conducted in (B), (D), (F), (H) and (J). *ns*, not significant; **p*<0.05, ***p*<0.01, ****p*<0.001, *****p*<0.0001.

**Supplementary Figure 6**


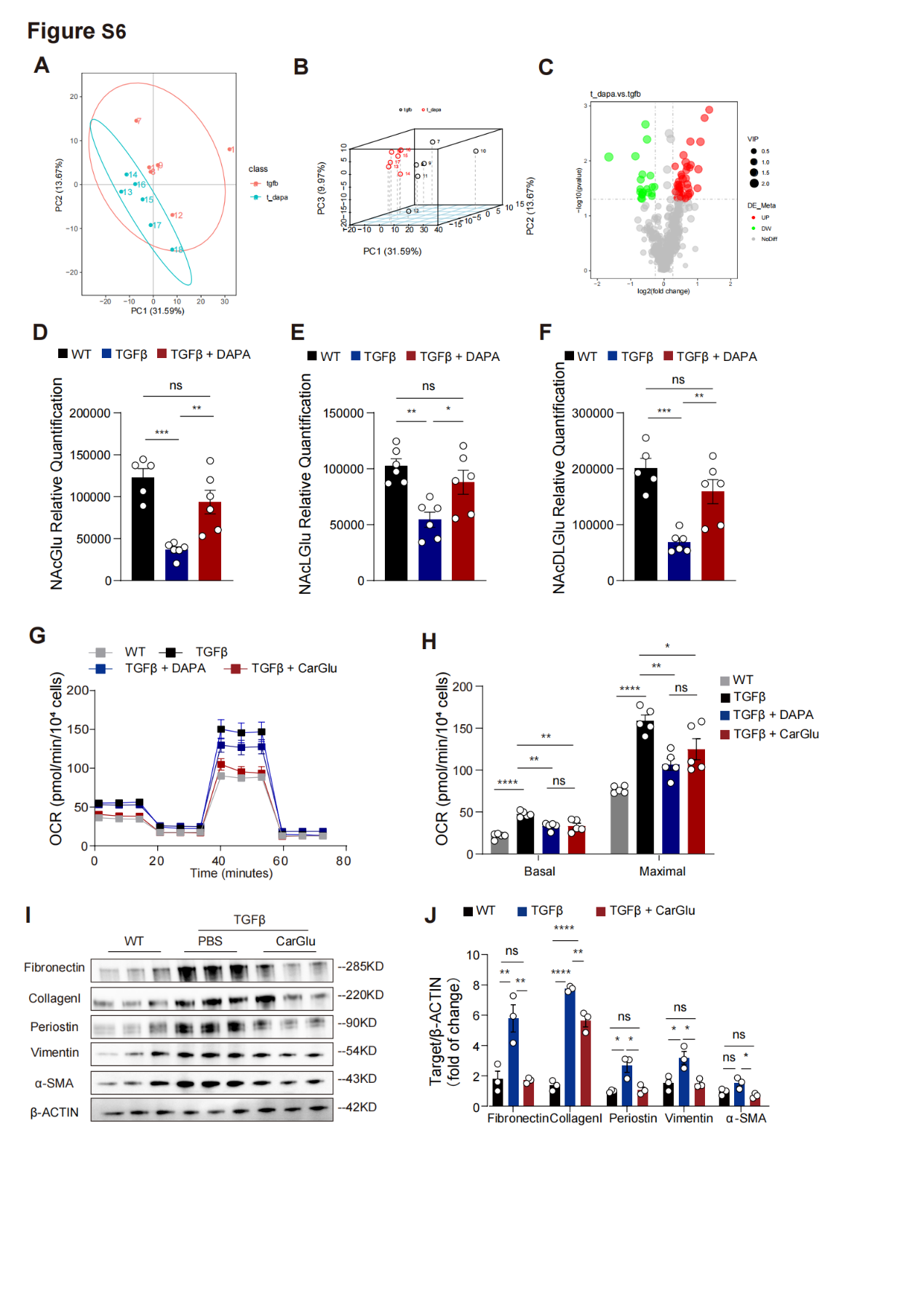


**Fig. S6. DAPA and CarGlu modulate fibroblast activation.**

(A-B) PCA (principal component analysis) of the TGF-β-stimulated fibroblasts received PBS or DAPA treatment for 24 hours. (C) Volcano map visualized the statistical results of differential metabolites. (D-F) Relative quantitative analysis of different structured NAcGlu from Quasi-Targeted Metabolomics. *n =* 5-6 in each group. *ns*, not significant; **p*<0.05, ***p*<0.01, ****p*<0.001. (G) OCR of fibroblast in live cell was recorded and analyzed using Seahorse XFe extracellular flux analyzer. (H) Quantitative analysis of basal and maximal respiration was plotted. *n =* 5 in DMEM group, *n =* 5 in TGF-β group, *n =* 5 in TGF-β+DAPA, *n =* 5 in TGF-β+CarGlu group. *ns*, not significant; *p<0.05, **p<0.01, ****p < 0.0001. (I) Immunoblots for fibrotic proteins were conducted in DMEM-treated NRCFs and TGF-β-stimulated NRCFs that received CarGlu treatment. (J) Quantitative bar graphs of fibrotic proteins; *n =* 3 in each group. *ns*, not significant; **p*<0.05, ***p*<0.01, *****p* < 0.0001. Among all statistical plots, the data are presented as the mean ± SEM. One-way ANOVA followed by Tukey’s post hoc multiple comparisons test was conducted in (D-F), (H) and (J). *ns*, not significant; **p*<0.05, ***p*<0.01, ****p*<0.001, *****p* < 0.0001.

**Supplementary Figure 7**


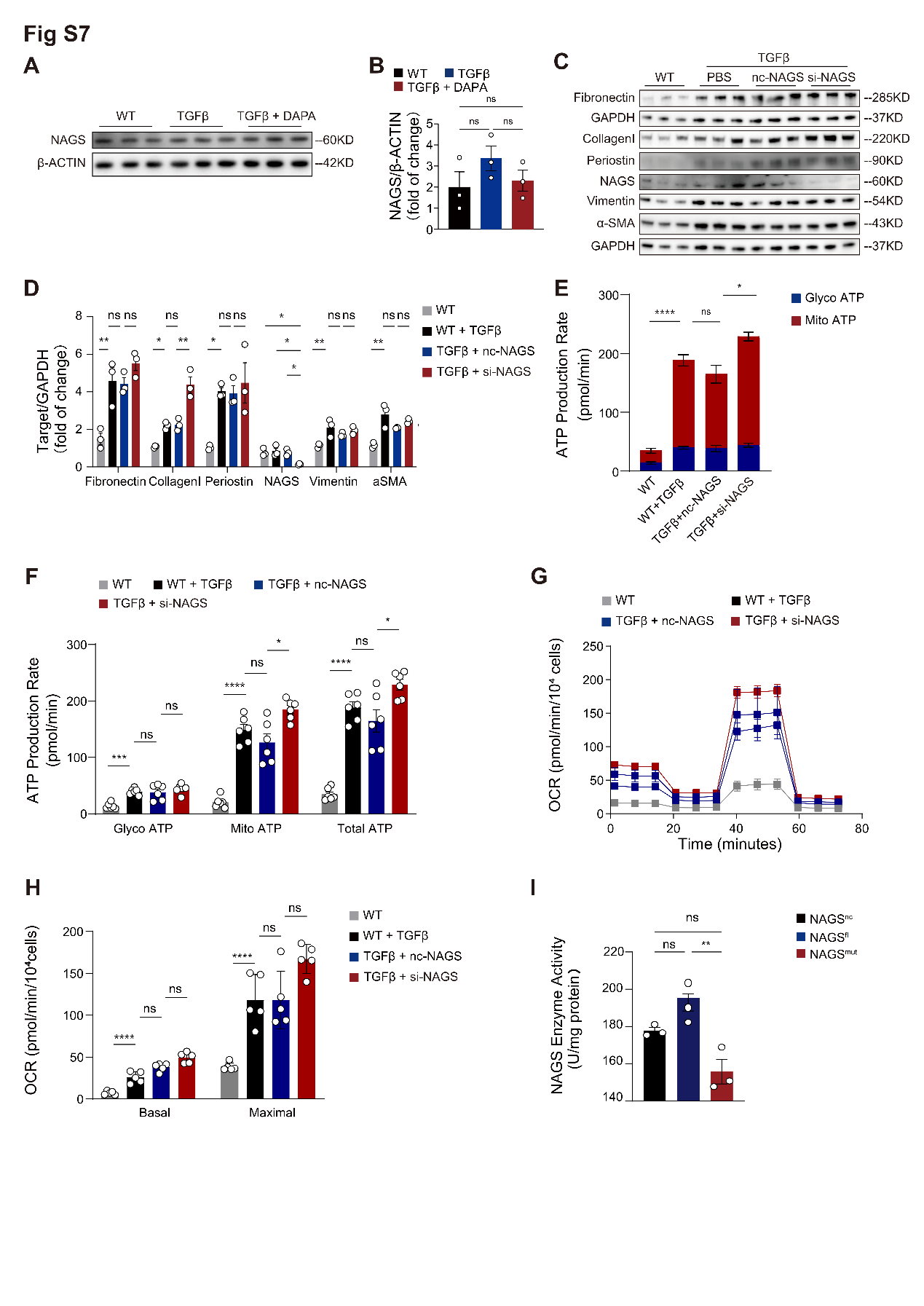


**Fig. S7. NAGS inhibition in fibroblasts.**

(A) Immunoblots for NAGS protein was conducted in DMEM-treated NRCFs and TGF-β-stimulated NRCFs that received DAPA. (B) Quantitative bar graphs of NAGS proteins; *n =* 3 in each group. *ns*, not significant. (C) Immunoblots for fibrotic and NAGS proteins were conducted in DMEM-treated NRCFs and TGF-β-stimulated NRCFs that were transfected with nc-NAGS or si-NAGS; *n =* 3 in each group. (D) Quantitative bar graphs of fibrotic and NAGS proteins; *n =* 3 in each group. *ns*, not significant; **p*<0.05, ***p*<0.01. (E) ATP production rate was detected by Seahorse XFe extracellular flux analyzer. Blue represents ECAR, and red represents OCR. The statistical results were derived from the mito ATP in each group, *ns*, not significant; **p* < 0.05, *****p* < 0.0001. (F) Quantitative analysis of glyco ATP and mito ATP is shown. *n =* 6 in each group. *ns*, not significant; **p*<0.05, ****p*<0.001, ****p<0.0001. (G) OCR of fibroblast in live cell was recorded and analyzed using Seahorse XFe extracellular flux analyzer. (H) Quantitative analysis of basal and maximal respiration was plotted. *n =* 5 in each group. *ns*, not significant; ****p<0.0001. (I) NAGS enzyme activity was detected in NRCFs infected with NAGS^nc^ or infected NAGS^fl^ or NAGS^mut^ lentivirus under TGF-β stimulation by Elisa kit. *n =* 3 in each group. *ns*, not significant; ***p*<0.01. Among all statistical plots, the data are presented as the mean ± SEM. One-way ANOVA followed by Tukey’s post hoc multiple comparisons test was conducted in (B), (D), (E), (F), (H) and (I). *ns*, not significant; **p*<0.05, ***p*<0.01, ****p*<0.001, *****p*<0.0001.

**Supplementary Figure 8**


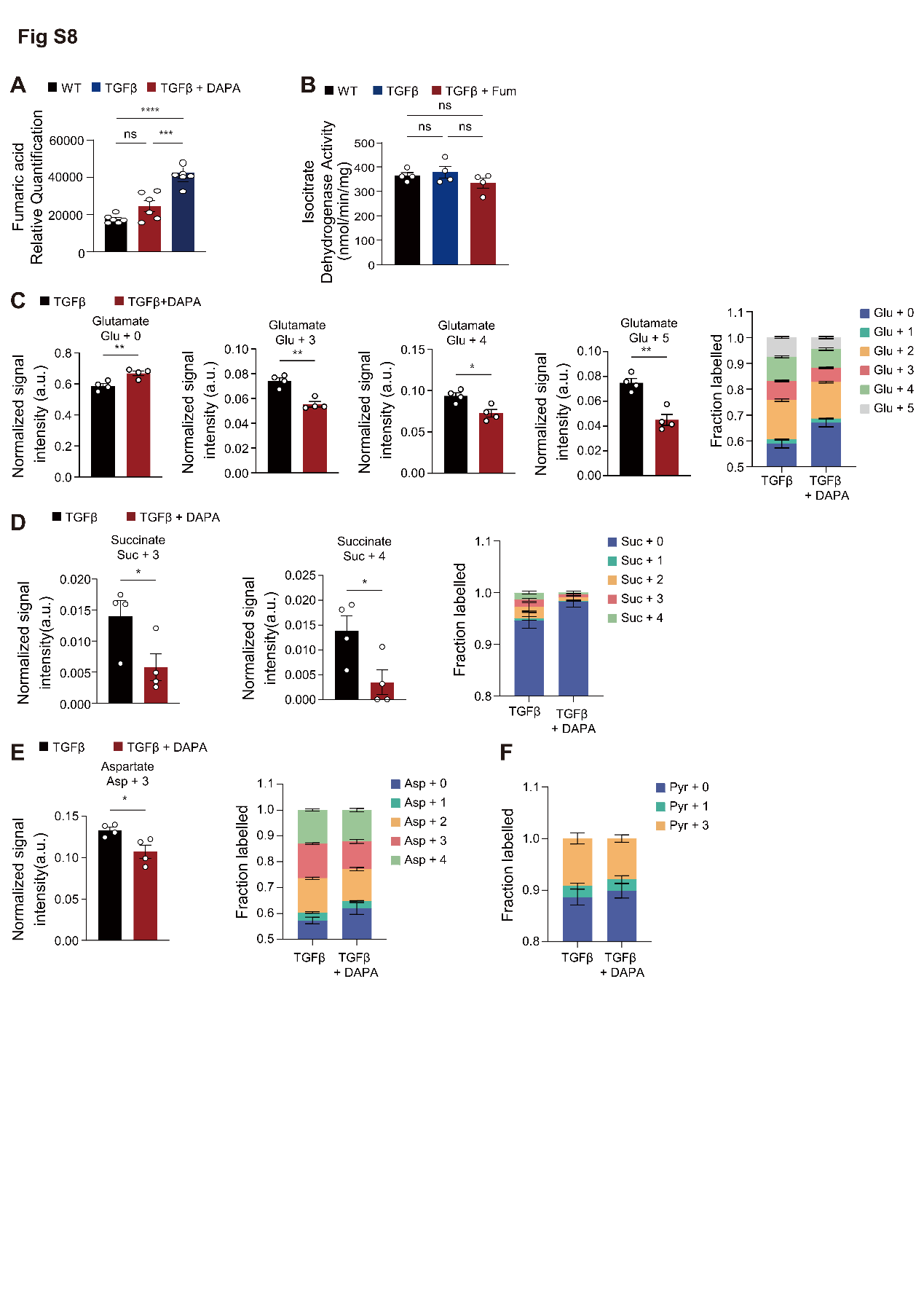


**Fig. S8. The effect of DAPA in regulating carbon source of intermediates in the TCA cycle.**

(A) Relative quantitative analysis of fumarate from metabolomics. *n =* 5*-*6 in each group. *ns*, not significant; ****p*<0.001, *****p*<0.0001. (B) Activities of key enzyme isocitrate dehydrogenase were measured in DMEM-treated and TGF-β-stimulated NRCFs treated with either DMSO or fumarate using an ELISA kit. *n =* 4 per group. *ns*, not significant. (C) Levels of glutamate were quantified using targeted liquid chromatography–tandem mass spectrometry (LC-MS), *n =* 4 in TGF-β and TGF-β + Fum groups. *ns*, not significant; **p*<0.05, ***p*<0.01. Level of Succinate (D), Aspartate (E) and Pyruvate (F) were quantified using targeted liquid chromatography-tandem mass spectrometry (LC-MS), *n =* 4 in TGF-β group, *n =* 4 in TGF-β+DAPA group. **p*<0.05. Among all statistical plots, the data are presented as the mean ± SEM. One-way ANOVA followed by Tukey’s post hoc multiple comparisons test was conducted in (A), (B). Two-tailed Student’s t-test was employed to compare two independent groups conducted in (C), (D) and (E). *ns*, not significant; **p*<0.05, ***p*<0.01, ****p*<0.001, ****p<0.0001.

**Supplementary Figure 9**


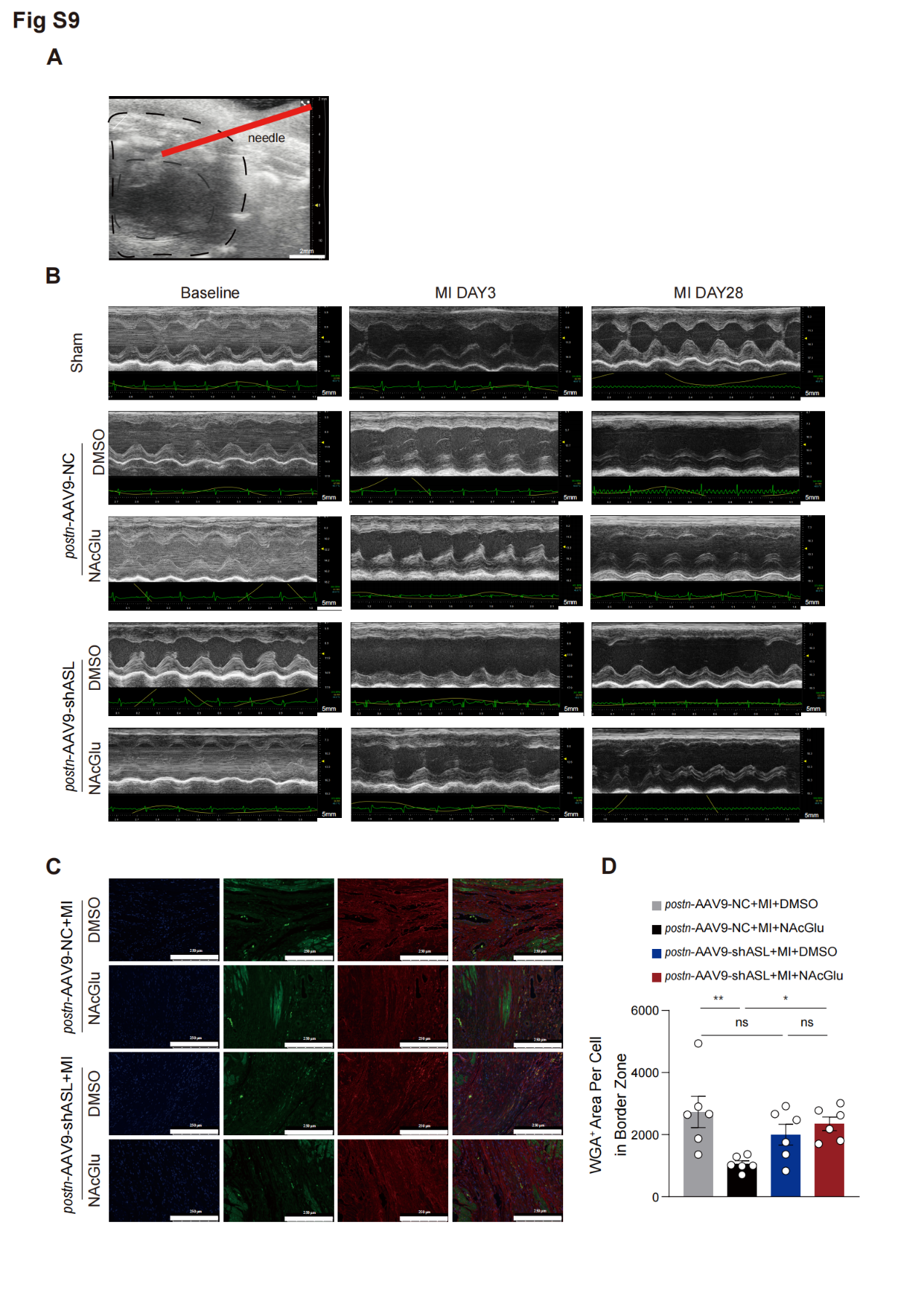


**Fig. S9. Knockdown of ASL and NAcGlu treatment modulates cardiac fibrosis post MI.**

(A) Representative images of B-mode echocardiography in myocardial injection under ultrasound guidance 2 weeks before MI surgery. (B) Representative images of M-mode echocardiography in each group at different time points. Sales bar = 2 mm. (C) Representative images of WGA immunostaining images from heart sections on rat from each experimental group, Scale bar = 250 μm. (D) Summary data on the proportion of WGA-positive area in each border zone; *n =* 6 in each group. ns, not significant; **p*<0.05, ***p*<0.01. Among all statistical plots, the data are presented as the mean ± SEM. One-way ANOVA followed by Tukey post hoc multiple comparisons test was conducted in (D). *ns*, not significant; **p*<0.05, ***p*<0.01.

**Supplementary Figure 10**

**
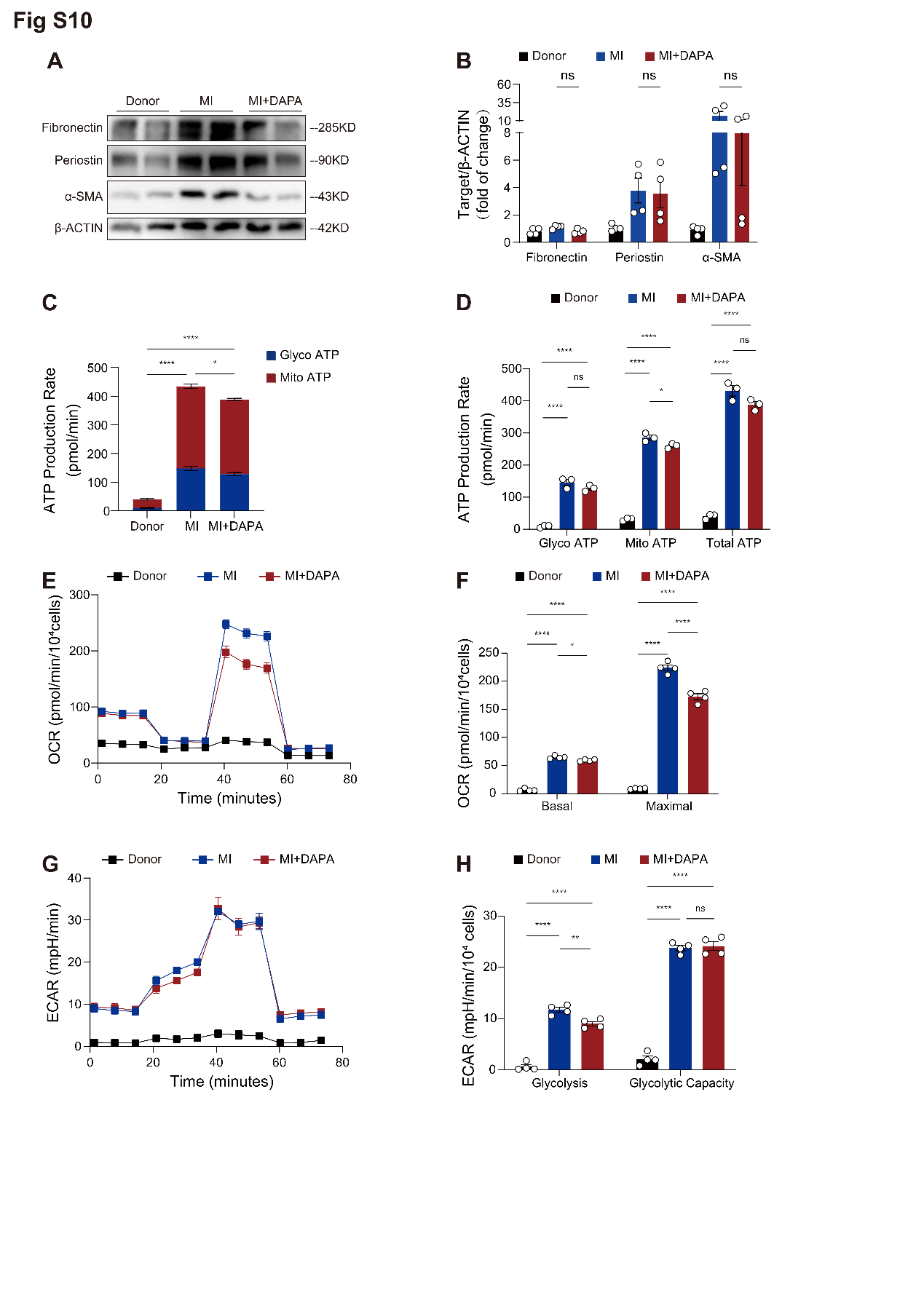
**

**Fig. S10. DAPA modulates the** **function of human cardiac fibroblasts (HuCFs).**

(A) Western-blot analysis of fibrotic protein in HuCFs isolated from organic lesions or brain-dead patients as control and MI-related heart failure and subsequently underwent heart transplantation, the HuCFs in MI+DAPA group were isolated from MI-related hearts and subsequently treated with DAPA. (B) Quantitative bar graphs of fibrotic proteins; *n = 4* in each group. *ns*, not significant. (C) ATP production rate measured using a Seahorse XFe extracellular flux analyser. The statistical results were derived from the mito ATP in each group, **p* < 0.05, *****p* < 0.0001. (D) Quantification of glyco ATP and mito ATP; *n =* 3 per group. *ns*, not significant; **p* < 0.05, *****p* < 0.0001. (E) The OCR of NRCFs was measured by a Seahorse XFe extracellular flux analyser. (F) Quantitative analysis of basal and maximal respiration: n = 4 per group. *p<0.05, ****p < 0.0001. (G) The ECAR of NRCFs was measured by a Seahorse XFe extracellular flux analyser. (H) Quantitative analysis of glycolysis and glycolytic capacity was plotted. *n =* 4 in each group. *ns*, not significant; ***p*<0.01, *****p* < 0.0001. Among all statistical plots, the data are presented as the mean ± SEM. One-way ANOVA followed by Tukey post hoc multiple comparisons test was conducted in (B), (C), (D), (F) and (H), *ns*, not significant; **p*<0.05, ***p*<0.01, ****p*<0.001, *****p* < 0.0001.

**Supplementary Table 1. Clinical characteristics and Blood Biochemistry Data**

|  | **Control** | **MI** |
| --- | --- | --- |
| Number  Gender(Male)  Age(Years old)  Height(cm)  Body weight(kg)  BMI(kg/m^2^)  Systolic blood pressure(mmHg)  Diastolic blood pressure(mmHg)  FBG(mmol/L)  LDL(mmol/L)  Total cholesterol(mmol/L)  Triglyceride(mmol/L) | 4  4  51.25±2.59  166.75±0.85  78.50±7.67  28.22±2.74  97±3.23  138.75±3.7  9.59±0.91  2.32±0.19  4.20±0.33  6.45±2.17 | 4  4  61±2.57  169±1.04  74.25±1.75  25.99±0.53  74±4.01  110±3.48  8.54±0.33  2.59±0.22  4.36±0.26  1.74±0.10 |
| BMI:body mass index; FBG:fasting blood-glucose; LDL,low density lipoprotein | | |

**Supplementary Table 2. Key antibodies table**

| Antibodies | Source | Identifier |
| --- | --- | --- |
| FITC anti-rat CD45 Antibody | BioLegend | Cat#202205; Clone:OX-1 |
| PE Mouse Anti-Rat CD31 Antibody | BD Pharmingen™ | Cat#555027; Clone:TLD-3A12 |
| Anti-Vimentin | Abcam | Cat#ab20346 |
| Anti-Ki67 | Abcam | Cat#ab16667 |
| Anti-Cardiac Troponin I | Abcam | Cat#ab56357 |
| Anti-CD3 | Abcam | Cat#16669 |
| Anti-CD68 | Abcam | Cat#125212 |
| Anti-Fibronectin | Abcam | Cat#ab2413 |
| Anti-Collagen I | Abcam | Cat#ab270993 |
| Anti-Collagen I | Abcam | Cat#ab34710 |
| Anti-Periostin | R&D | Cat#AF2955 |
| Anti-Periostin | Abcam | Cat#ab14041 |
| Anti-Vimentin | Abcam | Cat#ab92547 |
| Anti-α-SMA | Abcam | Cat#ab5694 |
| Anti--ASL | Abcam | Cat#ab201026 |
| Anti-NAGS | FineTest | Cat#FNab05539 |
| Anti-Phospho-p38MAPK  (Thr180/Tyr182) | Cell Signaling Technology | Cat#4092 |
| Anti-p38MAPK | Cell Signaling Technology | Cat#8690 |
| Anti-Phospho-p44/42 MAPK(Erk1/2) (Thr202/Tyr204) | Cell Signaling Technology | Cat#4370 |
| Anti-[p44/42 MAPK (Erk1/2)](https://www.cellsignal.com/products/primary-antibodies/p44-42-mapk-erk1-2-137f5-rabbit-monoclonal-antibody/4695) | Cell Signaling Technology | Cat#4695 |
| Anti-Phospho-AMPKα (Thr172) | Cell Signaling Technology | Cat#2535 |
| Anti-AMPKα | Cell Signaling Technology | Cat#2532 |
| Anti-Bcl-2 | SANTA CRUZ BIOTECHNOLOGY | Cat#sc-23960 |
| Anti-Bax | Cell Signaling Technology | Cat#2772 |
| Anti-Cleaved Caspase-3 | Cell Signaling Technology | Cat#9661 |
| Anti-Phospho-Stat3 (Ser727) | Cell Signaling Technology | Cat#9134 |
| Anti-Stat3 | Cell Signaling Technology | Cat#4904 |
| Anti-Phospho-Akt (Ser473) | Cell Signaling Technology | Cat#4060 |
| Anti-pan-AKT | Abcam | Cat#8805 |
| Anti-Phospho-GSK-3 beta (Ser9) | Cell Signaling Technology | Cat#5558 |
| Anti-GSK-3 beta (Ser9) | Cell Signaling Technology | Cat#12456 |
| Anti-GAPDH | Cell Signaling Technology | Cat#2118 |
| Anti-β-actin | Cell Signaling Technology | Cat#4967 |

**Supplementary Table 3. siRNA sequences for rat NAGS and ASL and homo ASL**

|  | sence(5’-3’) | antisence(3’-5’) |
| --- | --- | --- |
| Nags-Rat-1289 | CACGGUUACACUCGAUCUATT | UAGAUCGAGUGUAACCGUGTT |
| Nags-Rat-1127 | CCGAACUCUUCAGUAACAATT | UUGUUACUGAAGAGUUCGGTT |
| Nags-Rat-810 | CAGCAUUCCAAUCCUGUGUTT | ACACAGGAUUGGAAUGCUGTT |
|  | sence(5’-3’) | antisence(3’-5’) |
| Asl-Rat-840 | GCCCAAGGCAUCUUCAAAUTT | AUUUGAAGAUGCCUUGGGCTT |
|  | sence(5’-3’) | antisence(3’-5’) |
| Asl-Rat-1411 | CCAAGGAAUUCAACUUUGUTT | ACAAAGUUGAAUUCCUUGGTT |
|  | sence(5’-3’) | antisence(3’-5’) |
| Asl-Homo-1188 | CCACUGACCUUGCCUAUUATT | UAAUAGGCAAGGUCAGUGGTT |
| ASL-Homo-308 | GGCACCUUCAAACUGAACUTT | AGUUCAGUUUGAAGGUGCCTT |
| ASL-Homo-873 | CCAAGGAAUUCAGCUUCGUTT | ACGAAGCUGAAUUCCUUGGTT |
| ASL-Homo-190 | GGAUGUUCAAGGCAGCAAATT | UUUGCUGCCUUGAACAUCCTT |
