# Supplementary figures and images for: Urea cycle fumarate limits fibrosis post-myocardial infarction by reducing fibroblast mitochondrial adenosine triphosphate production
Source: Cardiovasc Res. 2026 May 28;122(10):1359–73. doi: 10.1093/cvr/cvag119 (PMC13355841; doi:10.1093/cvr/cvag119)

**Fig S3**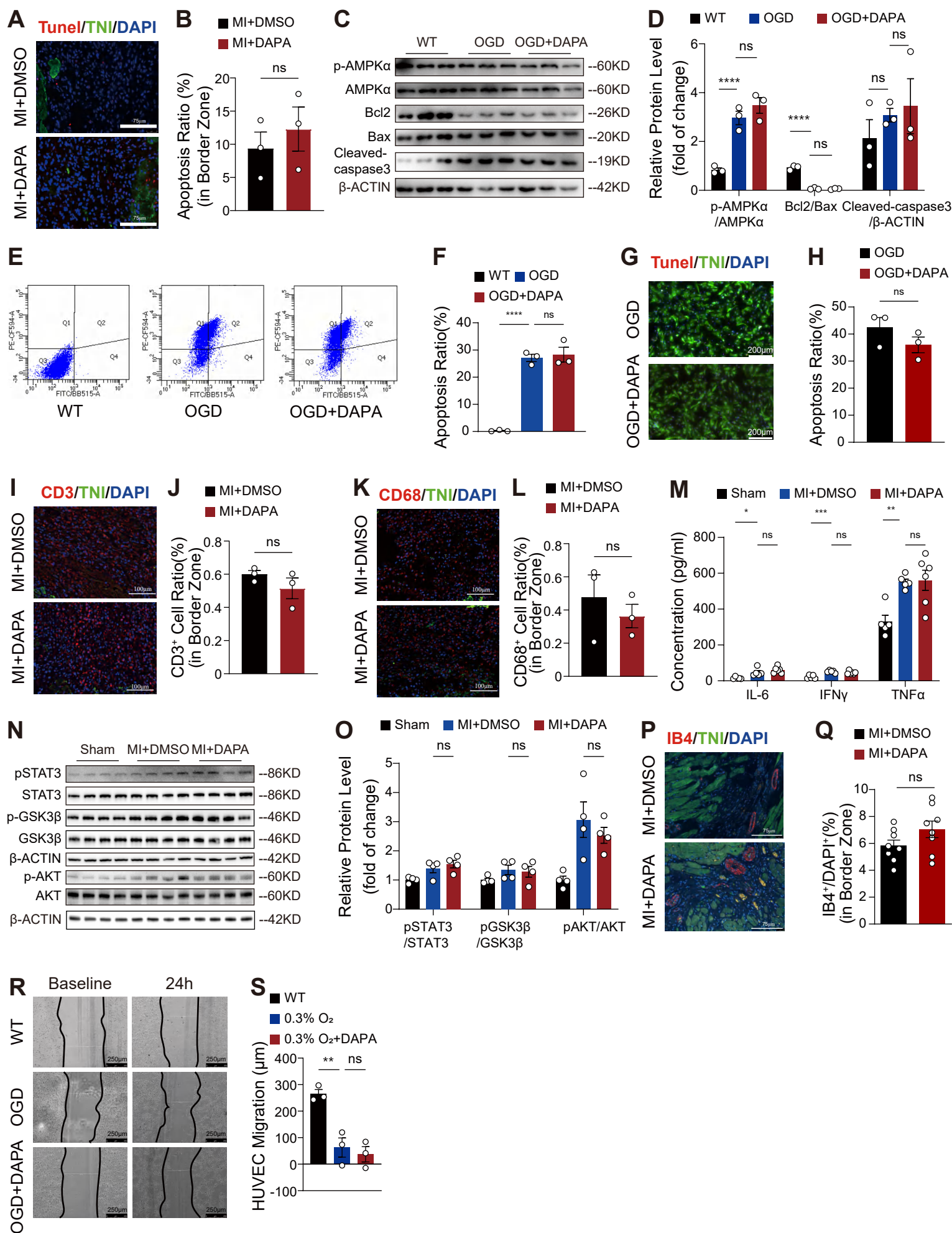

Supplement: cvag119_Supplementary_Data [file cvag119_supplementary_data.zip › S3 260309 R2.pdf]
